# Computational Repurposing of FDA-Approved Drugs as Candidate MMP2 Inhibitors with Putative MMP3 Cross-Activity

**DOI:** 10.3390/ijms27177756

**Published:** 2026-08-29

**Authors:** Saad Zekri, Nouhaila Ait Lahcen, Wissal Liman, Francesca Bianchini, Mehdi Oubahmane, Ismail Hdoufane, Driss Cherqaoui

**Affiliations:** 1 Laboratory of Molecular Chemistry, Faculty of Sciences Semlalia, Cadi Ayyad University, UCA, B.P. 2390, Marrakech 40000, Morocco; s.zekri.ced@uca.ac.ma (S.Z.); nouhaila.aitlahcen@ced.uca.ma (N.A.L.); wissallimane@gmail.com (W.L.); cherqaoui@uca.ma (D.C.); 2 Department of Experimental and Clinical Biomedical Sciences “Mario Serio”, Section of Experimental Pathology and Oncology, University of Florence, 50141 Florence, Italy; francesca.bianchini@unifi.it; 3 Mohammed VI Polytechnic University (UM6P), Hay Moulay Rachid, Ben Guerir 43150, Morocco

**Keywords:** Idiopathic pulmonary fibrosis, Matrix metalloproteinase-2, Matrix metalloproteinase-3, Pharmacophore Modeling, molecular docking, Molecular Dynamics simulation

## Abstract

Idiopathic pulmonary fibrosis (IPF) is a progressive interstitial lung disease characterized by excessive extracellular matrix remodeling and limited therapeutic options. Matrix metalloproteinase-2 is involved in extracellular matrix degradation and tissue remodeling, making it a relevant target for antifibrotic drug discovery. In this study, an MMP2-focused ligand-based pharmacophore model was developed and was applied to screen a curated FDA-approved drug library, leading to the identification of 83 pharmacophore-matching compounds. These compounds were subsequently prioritized through molecular docking against the catalytic site of MMP2, and the five best candidates were further evaluated by molecular dynamics (MD) simulations. Because of the biological relevance of MMP3 in pulmonary fibrosis, these five selected compounds were also profiled against MMP3 as a secondary target. Among them, Regorafenib (S1178) and Capmatinib (S2788) showed the most favorable cross-target profiles and were further supported by MD analysis. From these findings, S1178 and S2788 were proposed as promising MMP2-prioritized compounds with potential MMP3 cross-activity, warranting further experimental validation as candidate antifibrotic MMP modulators.

## 1. Introduction

Idiopathic pulmonary fibrosis (IPF) is one of the most severe and progressive types of interstitial lung diseases, and it is characterized by a poor prognosis and an irreversible reduction in lung function. Cigarette smoking, environmental pollution, and population aging are the main causes of its steadily increasing incidence in wealthy countries [1,2]. Early IPF lesions are usually caused by subclinical repetitive insults that damage the pulmonary epithelium. Repeated damage can result in endoluminal exudate accumulation, airspace collapse, and disruption of gas exchange [3]. At the dysfunctional alveolar-capillary interface, extracellular matrix (ECM) components are gradually and locally deposited by activated mesenchymal cells and fibroblasts. In this case, matrix metalloproteinases’ (MMPs) degradative function is crucial.

The MMP family consists of 26 zinc-dependent proteases, 24 of which are present in humans [4]. The ECM proteins that provide tissues structural integrity are broken down by these proteins [5]. In addition to degrading the ECM, MMPs are involved in numerous signaling pathways and have been implicated in the pathogenesis of several diseases, such as cancer, cardiovascular disorders, and inflammatory conditions [6,7]. Among them, MMP2 (gelatinase A) has received special interest since it can break down type IV collagen, which is a major component of basement membranes, along with other ECM proteins such as fibronectin, laminin, elastin, and type V collagen [5]. Together with MMP9 (gelatinase B), MMP2 constitutes part of the gelatinase subgroup. The aberrant overexpression of MMP2 has been linked to tumor growth, angiogenesis, invasion, and metastasis, with elevated levels reported in various cancers, including breast cancer and myeloid leukemia [8]. Beyond oncology, direct evidence from patients with pulmonary fibrosis supports MMP2 as a pathogenic mediator rather than a purely oncological target. In interstitial lung disease patients, both serum and bronchoalveolar lavage fluid (BALF) concentrations of MMP2 are significantly higher in fibrotic than in non-fibrotic cases, and immunofluorescence staining of fibrotic lung tissue from lung-transplant recipients shows spatial co-localization of MMP2 with denatured collagen within areas of fibrosis [9]. Earlier BALF studies in IPF patients reported elevated MMP levels linked to abnormal alveolar-capillary permeability and worse clinical outcome [10]. Consistent with this, explanted IPF lung tissue shows significantly higher MMP2 protein levels than non-fibrotic disease-control lung, although neither MMP2 deletion alone nor combined MMP2/MMP9 deletion prevented fibrosis in an adenoviral TGF-β1-driven mouse model, which indicates that MMP2 is not indispensable for fibrosis in this model and that other proteolytic or profibrotic pathways can sustain the response [11]. Mechanistically, MMP2 may disrupt alveolar basement membrane integrity, promote fibrocyte migration, and activate latent TGF-β1, thereby contributing to profibrotic signaling [12]. These findings support further investigation of MMP-2 as a candidate target within fibrotic remodeling. MMP3 (also known as stromelysin-1) degrades various components of the ECM, in particular type III, IV, X, and IX collagens, but also activates interstitial pro-enzymes, growth factors, and other matrix metalloproteinases. Thus, MMP3 has been recently identified as a key mediator of pulmonary fibrosis, contributing to the activation of proinflammatory cytokines and the induction of epithelial–mesenchymal transition (EMT), further exacerbating IPF disease progression. Together, MMP2 and MMP3 contribute to pathological ECM turnover, epithelial remodeling, and profibrotic signaling, supporting their investigation as complementary therapeutic targets in IPF [13,14].

Currently, after a diagnostic classification, the therapeutic approach for IPF focuses more on slowing the progression of the disease rather than on finding a cure. The two drugs now used as antifibrotic medicines are pirfenidone and nintedanib. These two drugs have been approved for the clinical use and have shown efficacy in reducing the rate of lung function decline. Nintedanib functions as a tyrosine kinase inhibitor that targets multiple growth factor receptors [15], whereas pirfenidone acts mainly through modulation of transforming growth factor-beta (TGF-β) signaling [16]. More recently, nerandomilast, an oral preferential phosphodiesterase-4B (PDE4B) inhibitor with combined antifibrotic and immunomodulatory effects, was approved for IPF after the phase 3 FIBRONEER-IPF trial showed that it significantly slowed the decline in forced vital capacity relative to placebo, both as monotherapy and on top of existing antifibrotic therapy, providing a third, mechanistically distinct treatment option [17]. The overall impact of the current treatments on patient survival, quality and life expectancy remains limited despite this advancement. Therefore, there is an urgent need to further clarify the molecular mechanisms underlying the IPF and also to identify novel therapeutic agents with an improved efficacy.

Importantly, although several other MMP family members such as MMP7, MMP8, and MMP9 have been implicated in IPF progression, the prioritization of MMP2 and MMP3 in this study is based on their complementary mechanistic roles within the fibrotic cascade. MMP2 acts primarily at the level of basement membrane degradation and fibroblast invasion, whereas MMP3 functions as an upstream activator of multiple pro-MMPs, including MMP7 and MMP9, thereby amplifying proteolytic and profibrotic signaling networks. This hierarchical positioning suggests that targeting MMP3 may indirectly modulate the activity of several downstream MMPs, while simultaneous inhibition of MMP2 directly limits matrix degradation and epithelial injury. While current antifibrotic therapies, including pirfenidone and nintedanib, primarily act through modulation of inflammatory and growth factor signaling pathways, they do not directly target extracellular matrix-degrading enzymes. Nevertheless, IPF is a multifactorial disease involving extensive redundancy among protease networks, and inhibition of MMP2 and MMP3 alone may not be sufficient to fully halt disease progression. However, accumulating experimental and clinical evidence supports their significant contribution to epithelial damage, ECM remodeling, and profibrotic signaling, indicating that their combined modulation could potentially attenuate key pathological processes in IPF [11,12]. In this context, dual inhibition of MMP2/MMP3 represents a mechanistically distinct approach that may complement existing therapies. Such a dual-targeting strategy may therefore provide a broader regulatory effect compared to single enzyme inhibition.

Within computational methods, the development of predictive models for virtual screening (VS) has been made possible by ligand-based pharmacophore modeling, which has been shown highly effective in identifying key chemical features common to bioactive molecules [18,19]. When combined with drug repurposing, which investigates FDA-approved drugs for novel therapeutic applications, this strategy becomes even more effective [20]. Because repurposed compounds already have established pharmacokinetic and toxicity profiles, this approach accelerates the process of discovery while minimizing safety concerns. To our knowledge, the dual MMP2/MMP3 repurposing potential of these candidates has not previously been investigated in IPF.

In this study, a ligand-based pharmacophore model was built based on a set of sulfonamide derivatives with reported inhibitory activity against MMP2. After validation, the model was used to screen an FDA-approved drug library to identify potential repurposing candidates. The selected drugs were docked against MMP2, and the most promising five compounds were further evaluated by molecular dynamics (MD) simulations to investigate the stability of the resulting protein–ligand complexes. Since MMP3 is also implicated in IPF-related ECM remodeling, the five compounds were subsequently profiled against MMP3 using molecular docking, followed by MD simulations analysis. In the context of IPF disease, this integrated computational workflow aimed to identify promising MMP2-prioritized compounds with potential MMP3 cross-activity for further optimization and experimental validation.

## 2. Results and Discussion

### 2.1. Ligand-Based Pharmacophore Modeling

**Figure 1:**
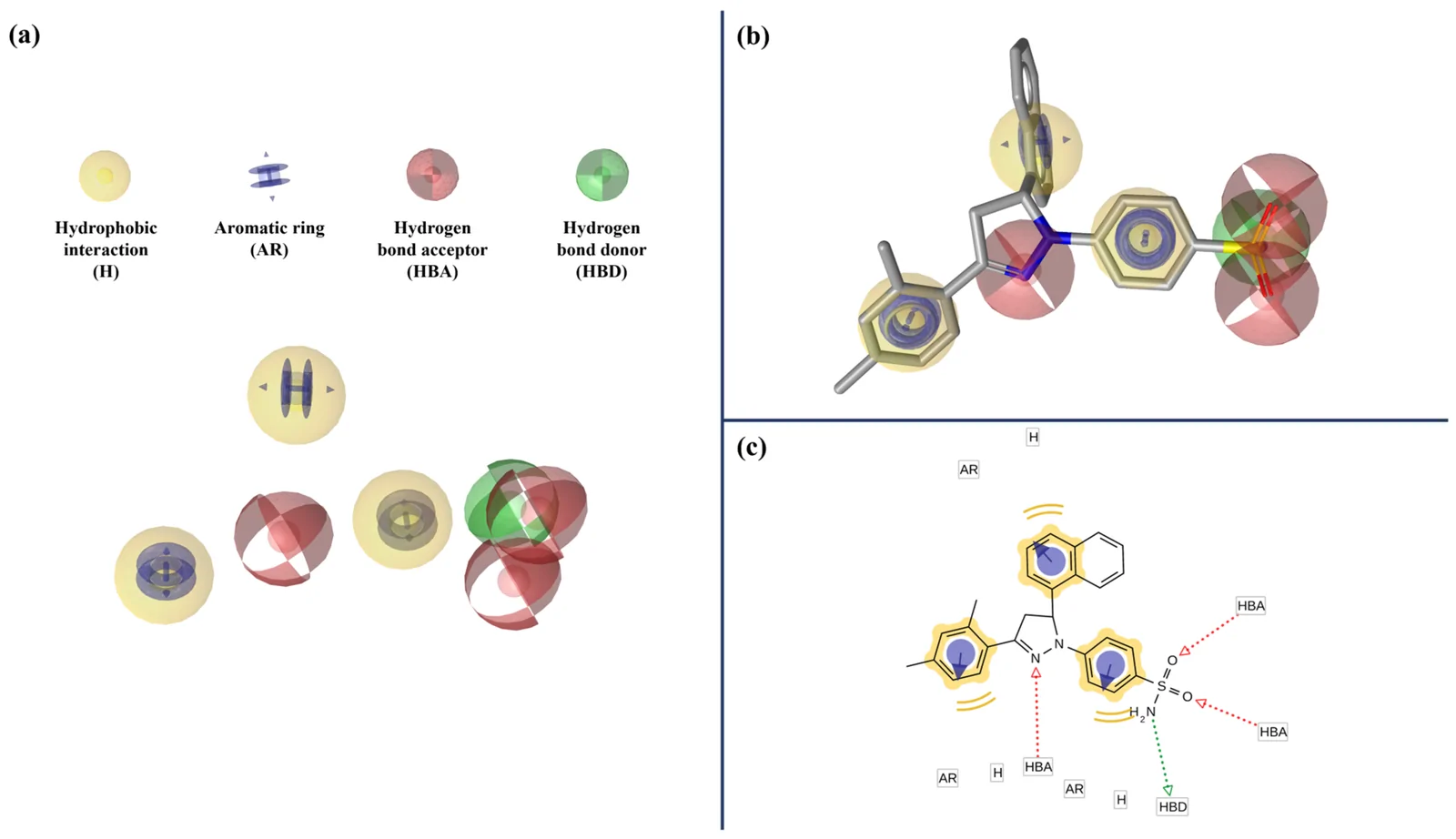
(**a**) Pharmacophore model features, (**b**) 3D conformational fit, and (**c**) ligand pharmacophore features for the lead compound (09).

A total of 10 pharmacophore models were built from a curated set of 25 sulfonamide derivatives with experimentally reported biological activities, followed by a rigorous internal validation using a random split (70/30), which preserved the activity distribution across training and test sets. The first model was selected due to its highest similarity score of 0.968. The selected model (Figure 1a) comprises 10 pharmacophoric features, including three hydrophobic (H) regions, three aromatic (AR) features, three hydrogen bond acceptors (HBA), and one hydrogen bond donor (HBD). Figure 1b,c displays the pharmacophore mapping of the lead compound (09). It was selected as the lead molecule because it exhibited the most potent MMP2 inhibitory activity among the 25 sulfonamide derivatives. The pharmacophore fit scores for the training and test compounds are reported in Table S1. These values varied from 98.64 to 108.90, which reflects that this model consistently captured the essential interaction features present across the dataset.

**Figure 2:**
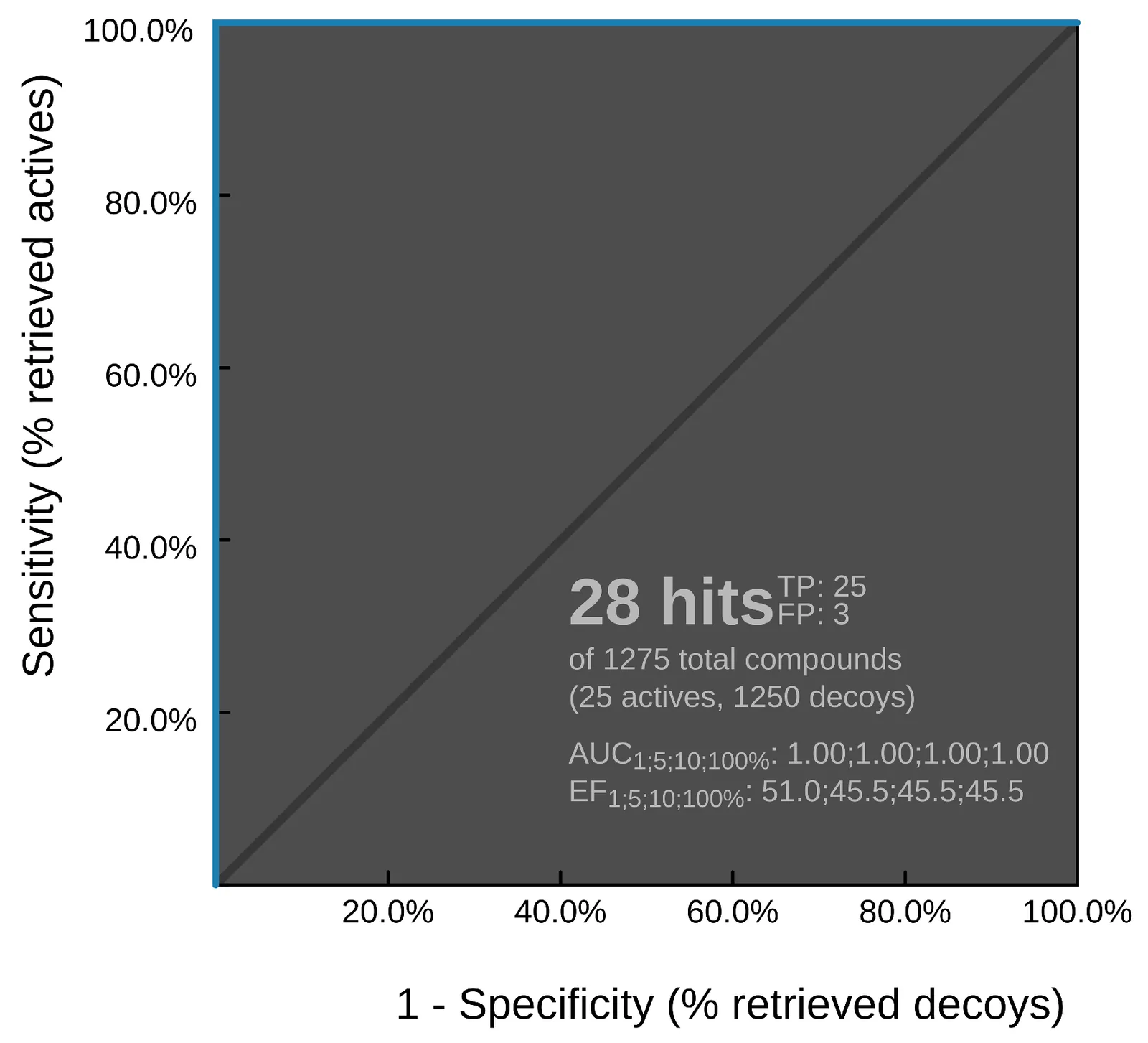
ROC curve analysis of the pharmacophore model for distinguishing actives from decoys.

To assess the reliability of the selected pharmacophore model, the VS of the active/inactive dataset was performed. This step is essential to determine whether the selected pharmacophore model could selectively extract known actives while excluding the inactives. As shown in Figure 2, the Receiver Operating Characteristic (ROC) curve analysis revealed a value of 1.00 which indicates an excellent discrimination between active and inactive compounds. The model also showed a sensitivity of 1.00, and a Specificity value of 0.997 confirming that all active compounds were successfully retrieved, and its strong ability to reject inactive compounds, respectively. Among the 28 compounds retrieved during validation, 25 were true positives (TPs) and only three were false positives (FPs), demonstrating a very low error rate. The goodness-of-hit (GH) score reaches 0.9174, which is above the accepted threshold of 0.6 for a robust pharmacophore model. In addition, the Enrichment Factor (EF) of 45.5 indicated a strong ability to concentrate active compounds within the retrieved hit list rather than selecting compounds randomly from the validation dataset. Together, the validation results confirmed that the selected pharmacophore model had strong discriminatory power, high enrichment capacity, and acceptable predictive reliability. A detailed summary of the validation parameters is provided in Table S2. Although such near-perfect metrics may raise concerns regarding potential overfitting, several points argue against this possibility. First, the model was not evaluated solely on the training data but on independent subsets and an external decoy set, reducing the likelihood of simple memorization. Second, similar high AUC values have been reported in the literature for well-defined pharmacophore models built on congeneric series, where consistent and strong interaction patterns dominate ligand recognition [21]. Third, the pharmacophore features were derived from chemically and biologically relevant interactions rather than purely statistical descriptors, supporting their mechanistic interpretability. Importantly, the benchmarking of the docking protocol yielded a lower AUC (0.869) on the same active/decoy set, indicating that the dataset does not inherently guarantee perfect classification and that the pharmacophore model provides genuine discriminatory power beyond trivial classification. Therefore, the observed performance is more plausibly attributed to the robustness of the identified pharmacophoric features within this chemical space rather than overfitting.

Therefore, it was applied for the VS of the FDA-approved drug library. To account for the structural diversity of FDA-approved drugs, compounds matching at least 70% of the 10 pharmacophoric features were retained (up to three features could remain unmatched). These features could increase the structural and scaffold diversity of the retrieved compounds. This relaxation was introduced to avoid restricting the screening to compounds matching all features of the original pharmacophore model and to facilitate the identification of chemically diverse scaffolds. The sensitivity, Specificity, and enrichment metrics reported above were obtained using the full 10-feature model prior to applying this relaxed screening criterion. The screening step identified 83 compounds that satisfied the essential pharmacophoric requirements, corresponding to about 2.7% of the curated library. These compounds were considered MMP2-oriented pharmacophore hits and were taken to molecular docking.

### 2.2. Molecular Docking and Virtual Screening

**Figure 3:**
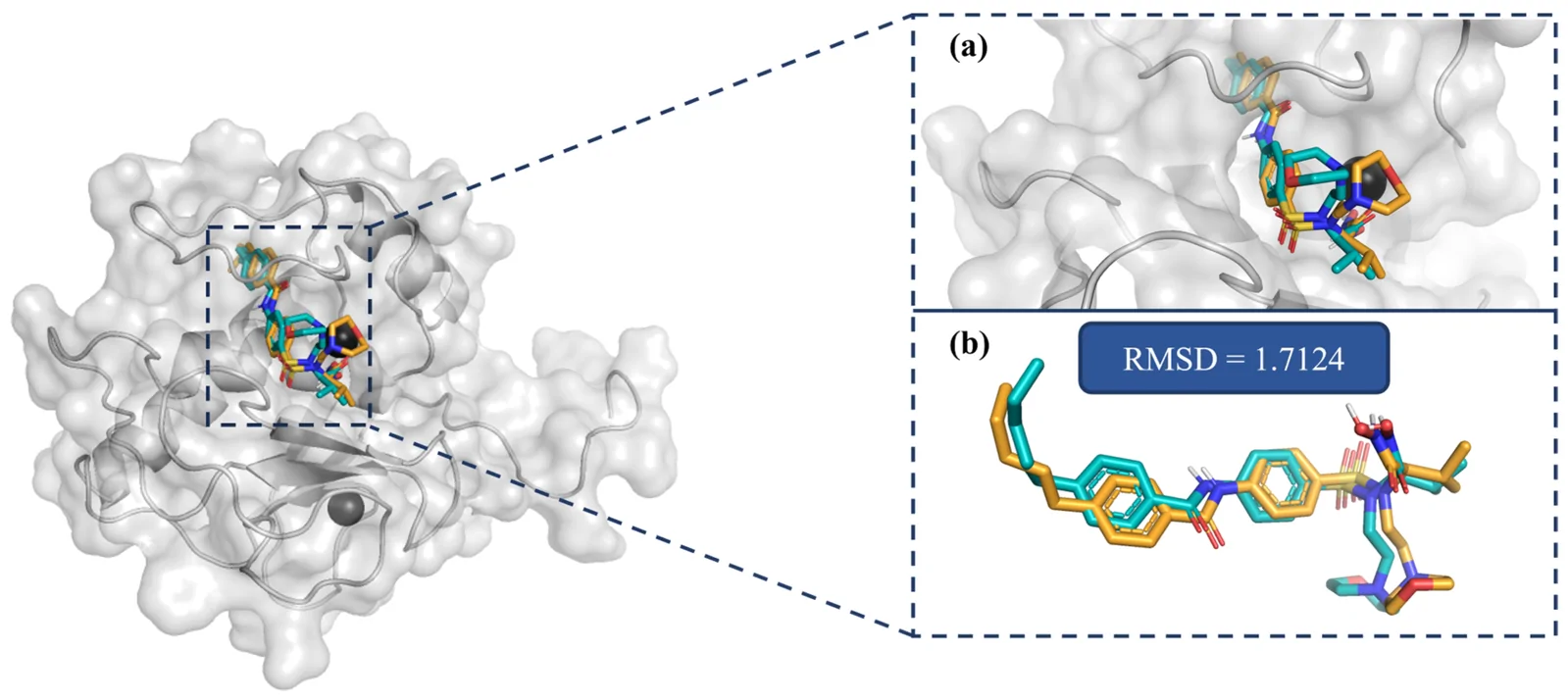
The docking protocol validation (**a**) zoomed view of the native ligand (orange) and docked pose (cyan) in the MMP2 active site. (**b**) Superposition of both conformations.

**Figure 4:**
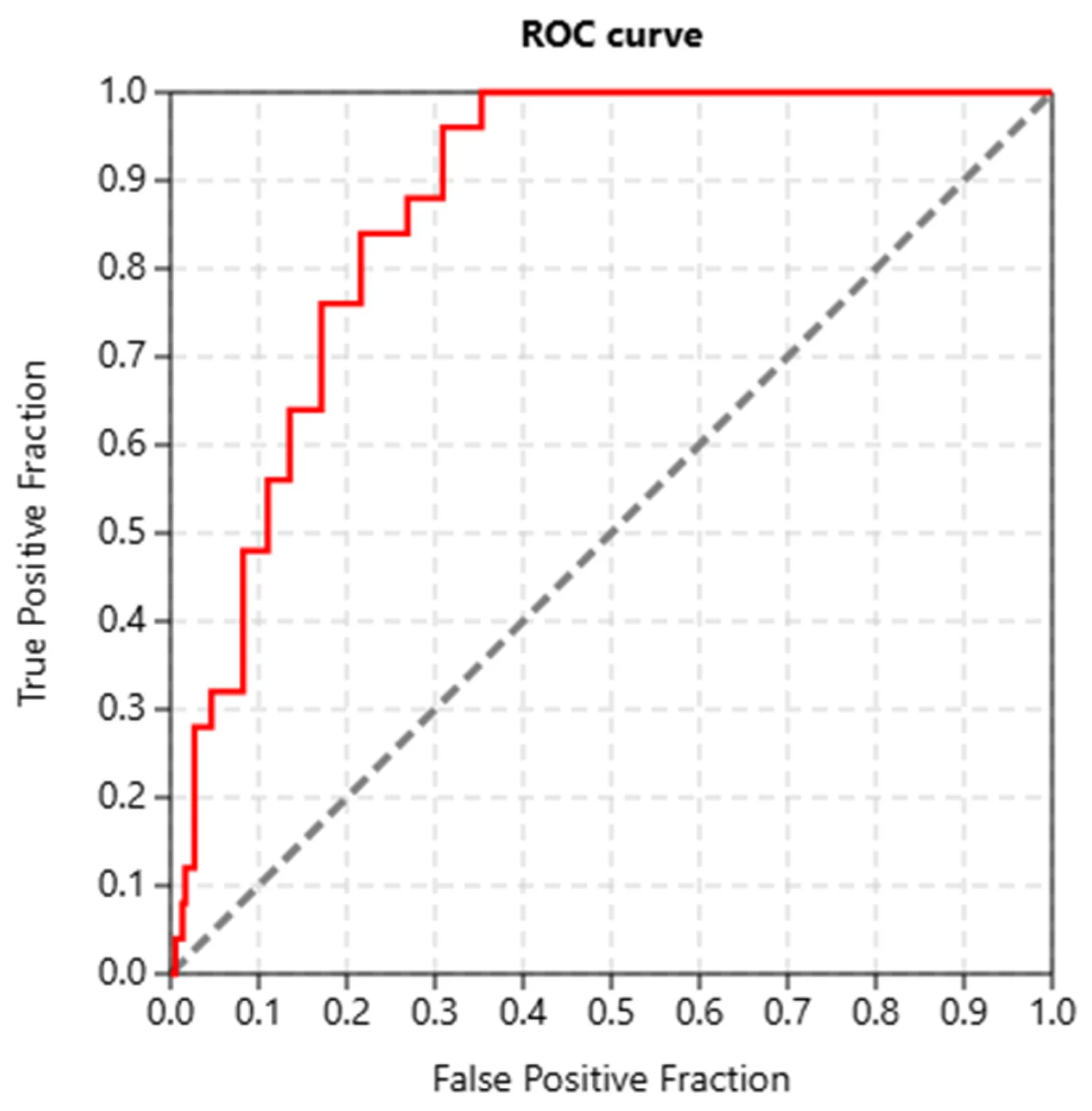
ROC curve of the docking protocol validation.

Molecular docking was used to rank and screen the 83 pharmacophore-derived hits inside the catalytic site of MMP2. The docking protocol was first checked by re-docking the 1HOV co-crystallized ligand (I52). The re-docked pose reproduced the crystallographic orientation with a Root Mean Square Deviation (RMSD) of 1.712 Å, below the 2.0 Å threshold commonly used for docking validation (Figure 3). The same protocol was then applied to the active/inactive dataset. The performance evaluation yielded an Area Under the ROC Curve (AUC) of 0.869, while the Total Gain (TG), Robust Initial Enhancement (RIE), and Boltzmann-Enhanced Discrimination of Receiver Operating Characteristic (BEDROC) values were 0.502, 4.977, and 0.301, respectively (Figure 4). TG reflects the discriminatory performance represented by the predictiveness curve, whereas RIE and BEDROC assess the early recognition of active compounds in the ranked list.

After validation, the 83 compounds were docked into MMP2. Compound 09, the lead molecule from the MMP2 inhibitor dataset, was used as the main reference. Compounds with docking scores better than compound 09 were retained. Five molecules met this criterion: S1178, S7397, S2788, S1005, and S2807. These correspond to the FDA-approved generic drugs Regorafenib (S1178), Sorafenib (S7397), Capmatinib (S2788), Axitinib (S1005), and Dabrafenib (S2807). S1178 gave the best docking score (−10.1 kcal/mol), followed by S7397 (−10.0 kcal/mol), S2788 (−9.8 kcal/mol) and both S1005 and S2807 with a score of −9.4 kcal/mol. These scores were more favorable than those of compound 09 (−9.3 kcal/mol) and the co-crystallized ligand I52 (−8.3 kcal/mol). The docking scores, structures, and pharmacophore fit values are summarized in Table 1 and Table S3.

**Figure 5:**
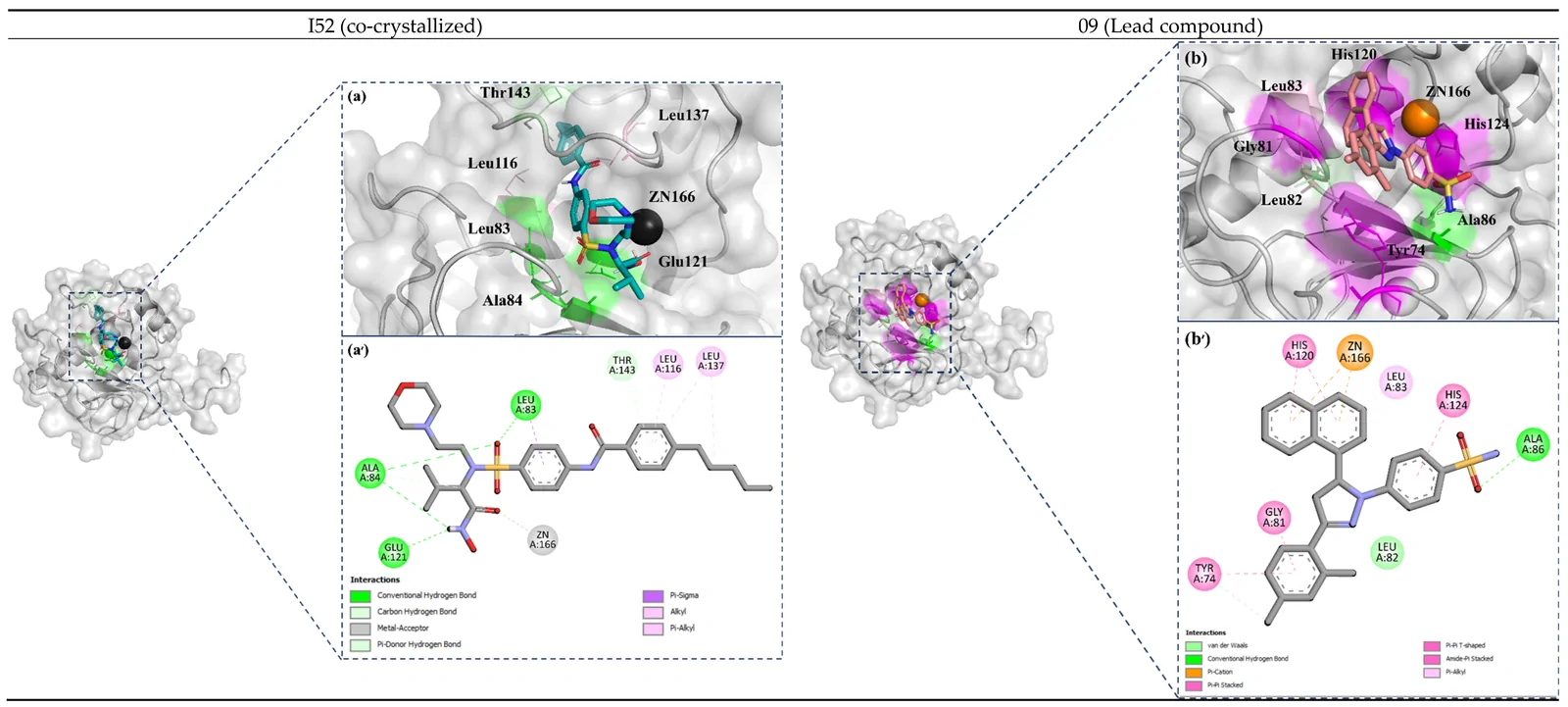

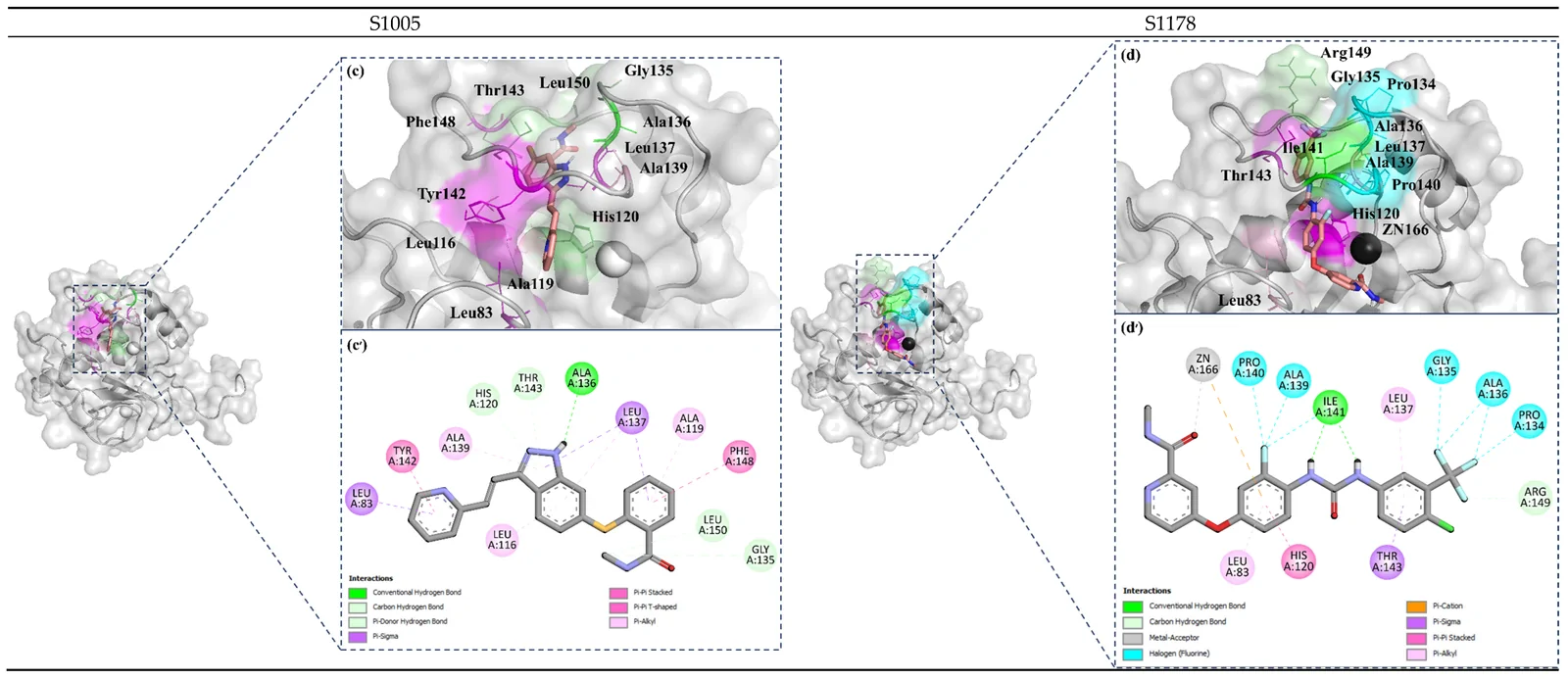

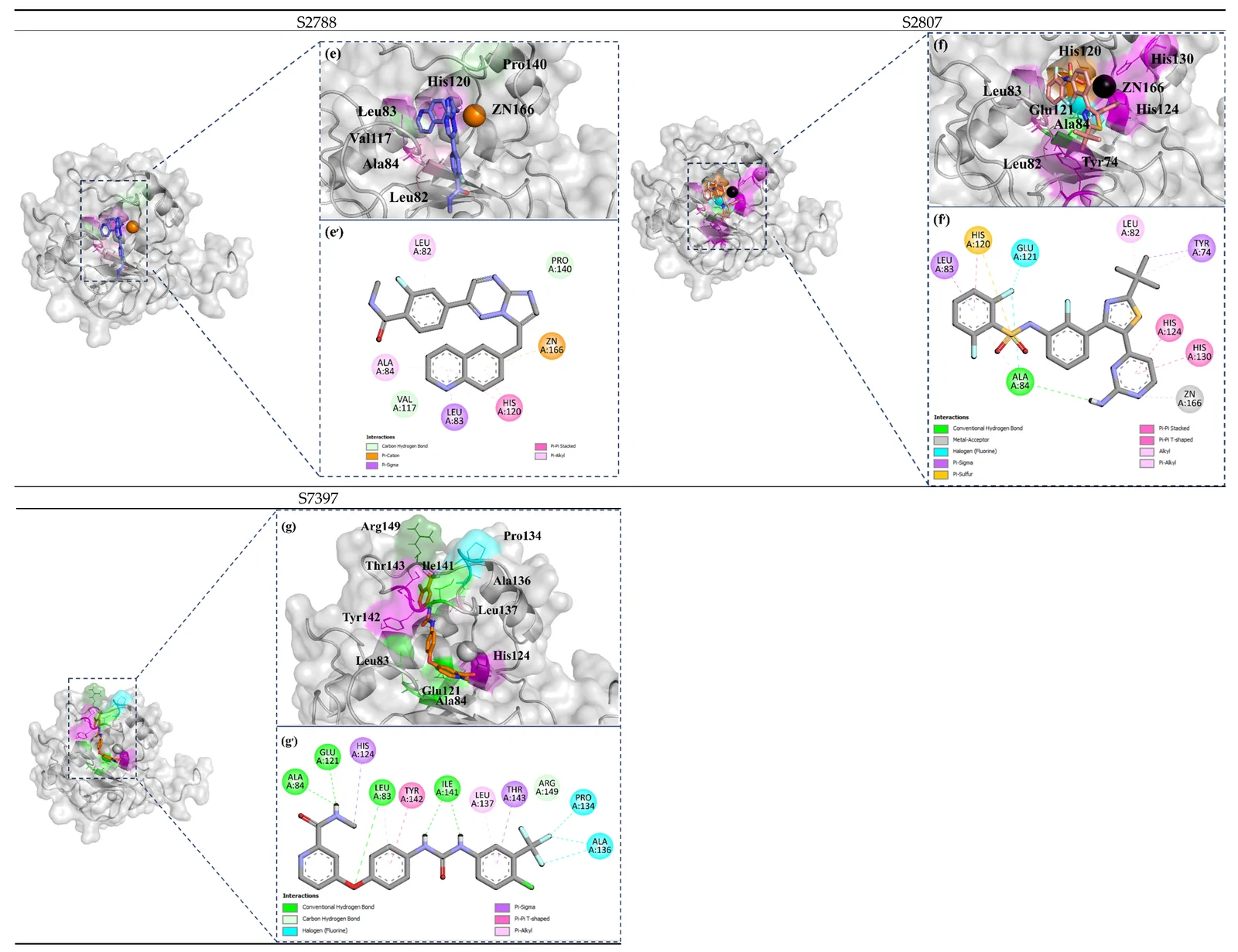
(**a**–**g**) 2D and (**a’**–**g’**) 3D interaction representations of the five selected FDA drugs, lead and co-crystallized compounds within the active site of MMP2 (1HOV).

To investigate the ligand–protein interaction profiles of the five selected compounds within the active site of MMP2, both 2D and 3D representations were investigated (Figure 5). The lead compound (09) established a strong binding profile within the MMP2 active site. It formed a conventional hydrogen bond with Ala86, electrostatic contact with Zn166, and hydrophobic interactions with His120, His124, Tyr74, and Leu83. The co-crystallized ligand (I52), in contrast, exhibited a broader hydrogen bonding network involving Leu83, Ala84, and Glu121. It also showed a metal–acceptor interaction with Zn166, together with π-donor and hydrophobic contacts with Leu116 and Leu137. When compared with I52 and 09, the selected FDA-approved compounds displayed interaction patterns that both mimicked and extended the binding features of the lead and co-crystallized compounds (I52 and 09). S1005 formed five hydrogen bonding interactions, resembling I52 in forming multiple polar contacts with Ala136, Gly135, Leu150, His120, and Thr143. It also shared hydrophobic contacts with His120 and Tyr142 similar to compound 09. This patter suggests a balanced combination of polar and aromatic stabilization. In addition, S1005 was stabilized by several hydrophobic interactions with Leu83, Leu116, Ala119, His120, Leu137, Ala139, Tyr142, and Phe148. However, it did not display any direct interaction with Zn166, indicating that its binding mode is mainly supported by hydrogen bonding and hydrophobic stabilization rather than zinc-directed recognition. S1178, the top-ranked compound, reproduced the metal coordination with Zn166 observed in both 09 and I52. It also introduced extensive halogen interactions with Pro134, Gly135, Ala136, Ala139, Pro140, and Ile141, which were absent in the references and likely contributed to its superior binding affinity. This compound formed three hydrogen bonds involving Ile141 and Arg149, a metal–acceptor interaction with Zn166, and an additional π-cation interaction with Zn166. It was further stabilized by hydrophobic interactions with Thr143, His120, Leu83, and Leu137. S2788 formed two hydrogen bonds with Pro140 and Val117, together with an electrostatic interaction with Zn166. It also formed several hydrophobic contacts with Leu82, Leu83, Ala84, and His120. S2807 resembled the co-crystallized ligand by engaging in a conventional hydrogen bond with Ala84, a metal–acceptor interaction with Zn166, and halogen interactions involving Ala84, Glu121. Moreover, S2807 displayed hydrophobic contacts with Leu83, Tyr74, His120, His130, His124, Leu82, and Ala84. Finally, S7397 displayed seven hydrogen bonds (Leu83, Ile141, Ala84, Glu121, and Arg149). It also formed three halogen interactions via Pro134 and Ala136, and hydrophobic contacts with Leu83, His124, Leu137, Tyr142, and Thr143. Unlike S1178, S2788, and S2807, S7397 showed no direct interaction with Zn166. The detailed docking interactions of the top five selected compounds, along with 09 and I52, are summarized in Table S4. According to earlier research, strong MMP2 inhibitors usually bind inside the catalytic domain by a combination of zinc chelation, hydrogen bonding with key residues such as Leu83, Ala84, Glu121, and π-stacking with His120. This combination is characteristic of high-affinity non-covalent MMP2 binders [22,23]. In accordance with this reported pharmacophoric pattern, the selected five FDA-approved compounds identified in this study recapitulated several canonical interactions, while additionally introducing stabilizing contributions such as halogen bonding and extended π-based contacts. In particular, S1178 and S2807 maintained direct metal–acceptor interactions with Zn166, while S2788 formed a π-cation electrostatic interaction with Zn166. In contrast, S1005 and S7397 lacked interaction with Zn166 but retained relevant MMP2 binding features through extensive hydrogen bonding, hydrophobic, and halogen contacts. These enriched interaction fingerprints align with recent evidence indicating that MMP2 active site stabilization is maximized when metal chelation is complemented by secondary noncovalent forces expanding into peripheral pockets of the S1′ region [22]. In summary, the selected FDA-approved compounds preserved key elements of the MMP2 binding pattern through distinct but complementary interaction profiles. S1178, S2788, and S2807 showed Zn166-associated interactions, supporting their ability to mimic the metal-directed recognition commonly observed in potent MMP2 inhibitors. In contrast, S1005 and S7397 lacked direct interaction with Zn166 but compensated through multiple hydrogen bonds and extensive hydrophobic contacts. These findings suggest that the five selected compounds may stabilize the active site of MMP2 through different non-covalent mechanisms. S1178, S2788, and S2807 showed stronger zinc-related binding features, whereas S1005 and S7397 contributed mainly through secondary polar, hydrophobic, and halogen interactions.

Based on the MMP2 docking results, MD simulations were performed to evaluate the stability of the interactions between MMP2 and the five selected compounds. Following the production runs, several structural and dynamic parameters, RMSD, RMSF, Rg, and SASA, were calculated to assess the protein–ligand stability (Table 2). The apo protein, the MMP2-I52 co-crystallized complex, and the MMP2-09 lead compound complex were used as comparative systems.

### 2.3. Molecular Dynamics Analysis of MMP2 Complexes

**Figure 6:**
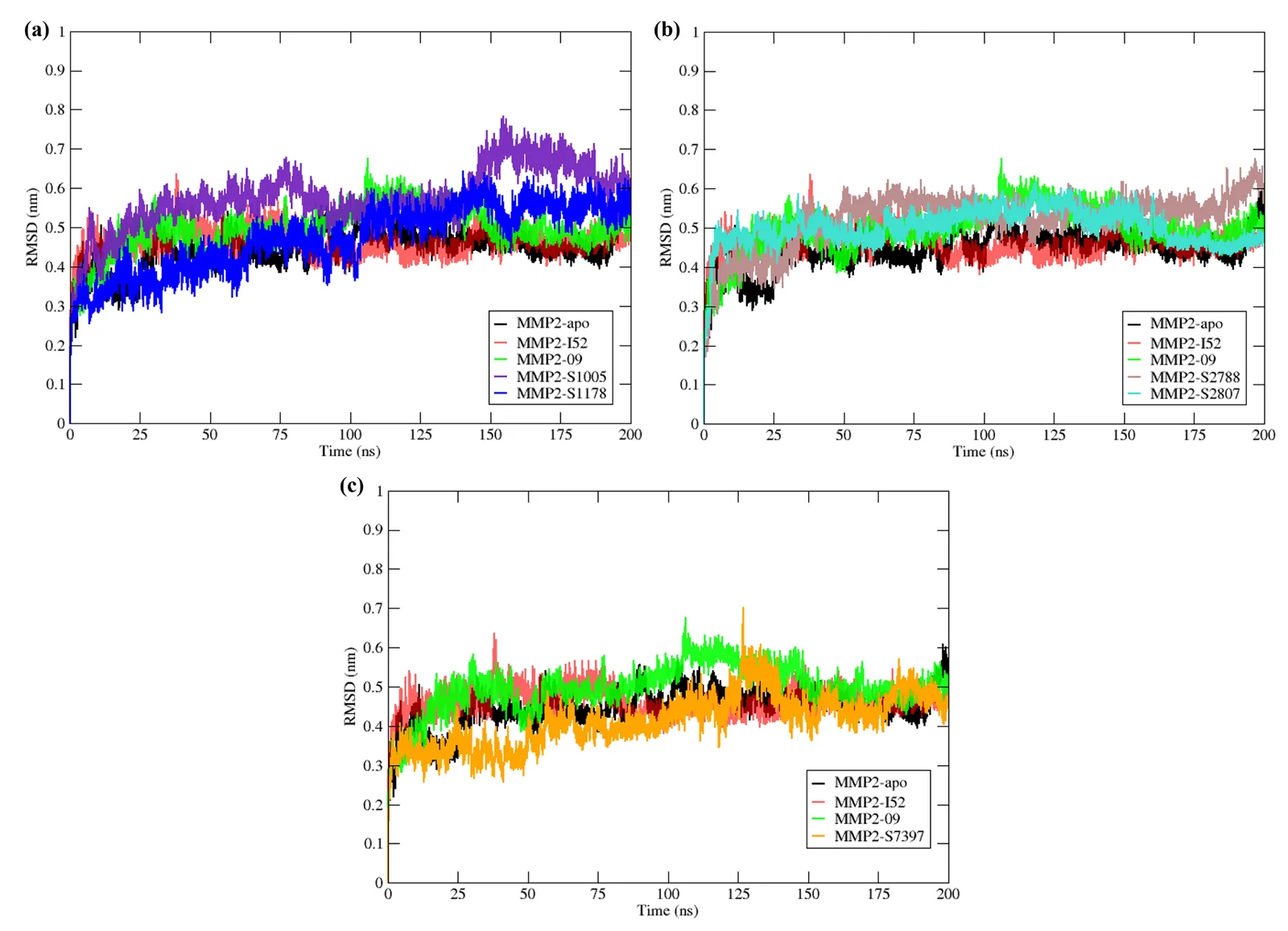

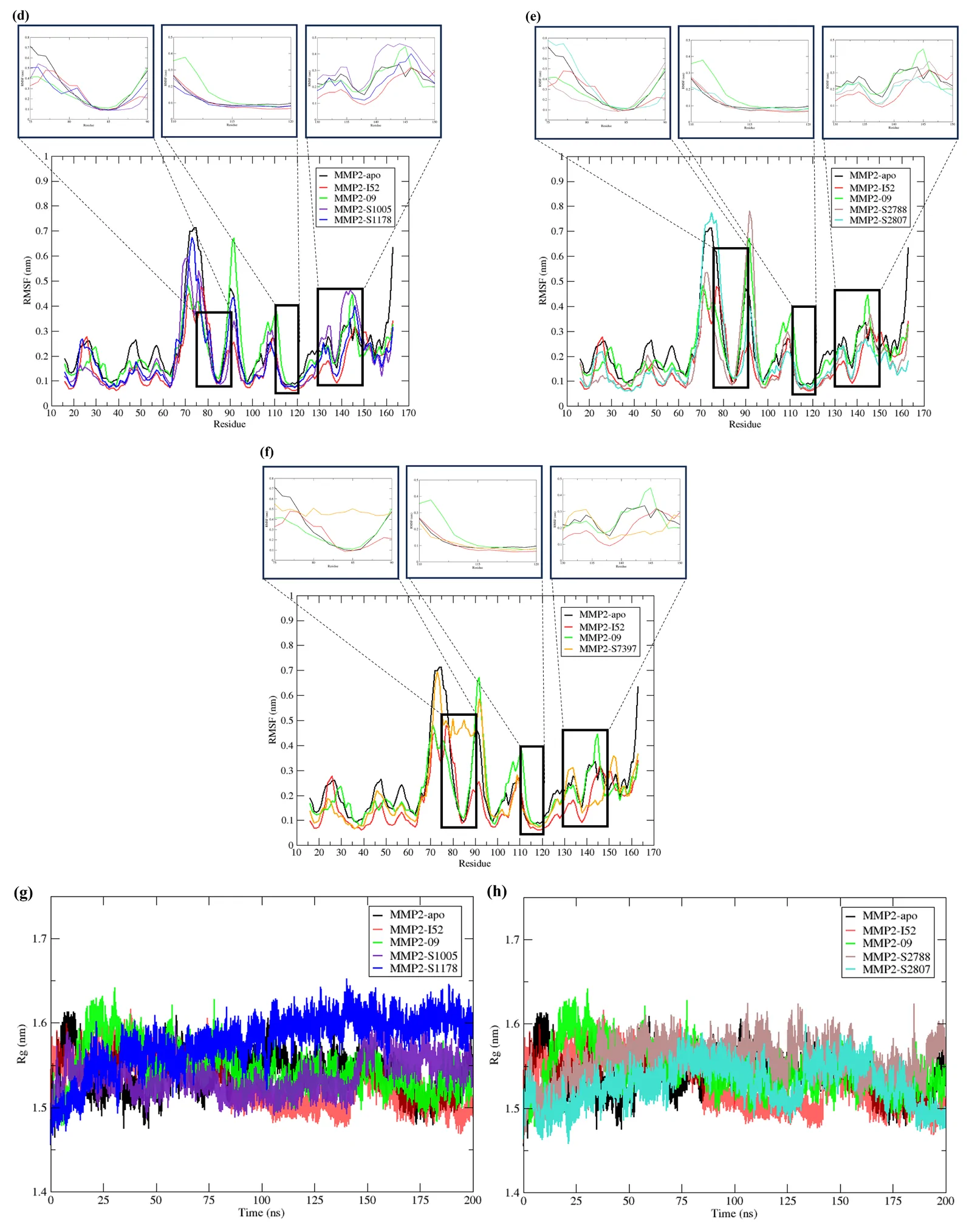

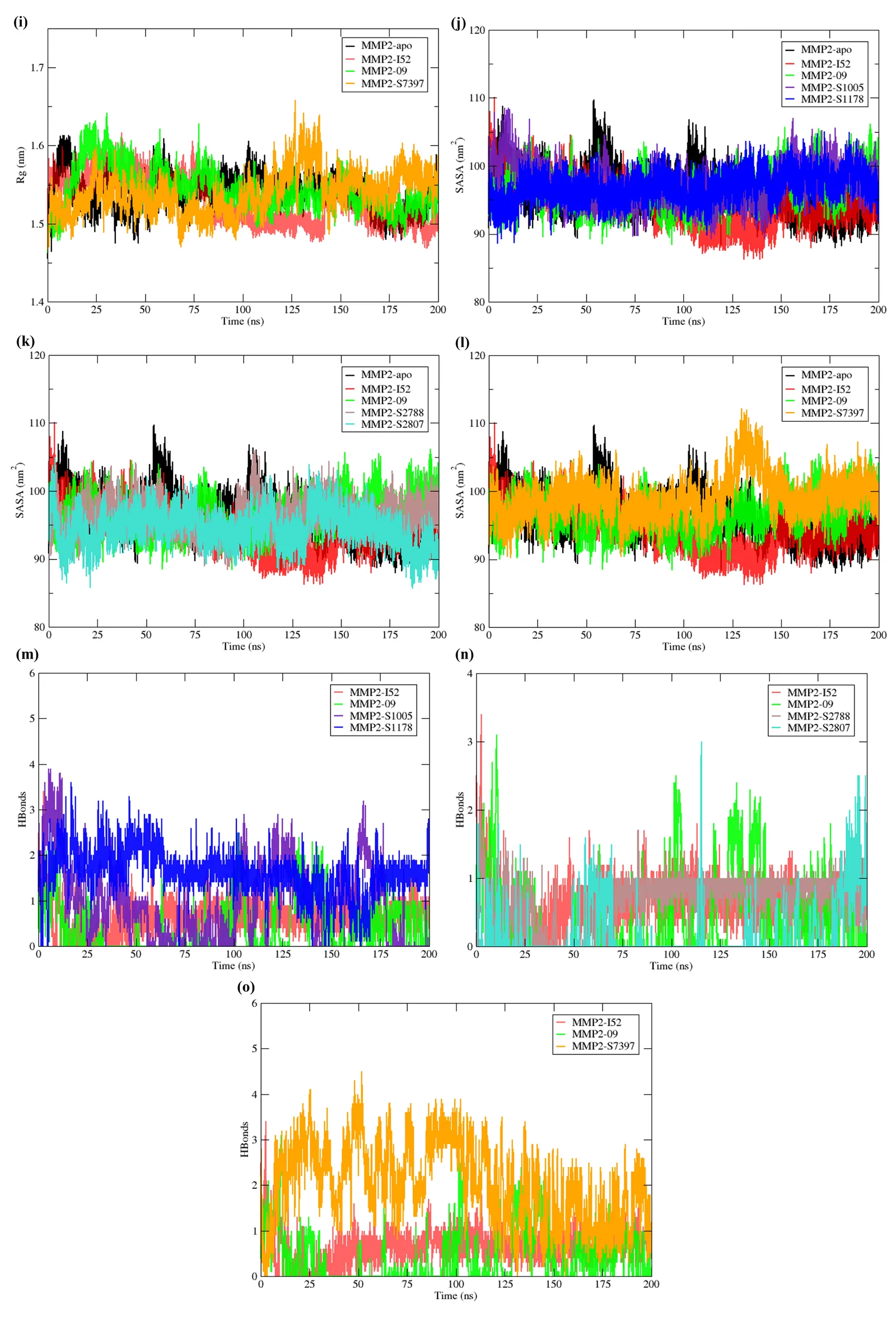
RMSD (**a**–**c**), RMSF (**d**–**f**), Rg (**g**–**i**), SASA (**j**–**l**) and HB (**m**–**o**) plots of co-crystallized ligand I52, lead compound 09, S1005, S1178, S2788, S2807, and S7397.

Backbone RMSD was first analyzed to compare the relative structural stability of the apo form of MMP2 and the ligand-bound systems. In MD simulations, RMSD measures the time-dependent deviation of the simulated structure from a reference conformation and is used to evaluate the overall structural stability of the system. As shown in Figure 6a–c and Table 2, the RMSD of the apo form of MMP2 initially fluctuated between 0.30 and 0.45 nm during the first 30 ns, after which it stabilized at around 0.43 nm, except for a slight increase to 0.50 nm in the final 10 ns. For the MMP2-I52 co-crystallized ligand complex, the RMSD was maintained between 0.40 and 0.60 nm during the first 30 ns and subsequently stabilized within 0.45–0.50 nm until the end of the trajectory. The lead compound complexed with MMP2 (MMP2-09) exhibited a rapid RMSD increase in the first 20 ns, reaching 0.40–0.50 nm, followed by stabilization. Over the simulation, this system remained equilibrated with fluctuations mainly within 0.45–0.60 nm, with occasional spikes slightly above 0.60 nm, suggesting overall conformational stability with minor structural variations. Among the newly identified hits, the RMSD profiles indicated different degrees of backbone adjustment rather than a single uniform behavior. The complex of MMP2-S2788 exhibited an initial sharp rise from 0.20 to 0.40 nm during the first 20 ns, consistent with structural relaxation upon ligand binding. After 30 ns, the system stabilized, with RMSD values ranging from 0.45 to 0.55 nm, except for a final increase to 0.60 nm during the last 10 ns. The MMP2-S2807 complex showed an RMSD increase during the first 20 ns, peaking at 0.50 nm, and remained stable between 0.45 and 0.55 nm until 120 ns. A slight increase was observed around 125 ns, but the system subsequently stabilized during the last 50 ns with RMSD values between 0.45 and 0.50 nm. The MMP2-S1005 and MMP2-S1178 complexes also remained within the same general RMSD range observed for the reference systems, although S1005 displayed the highest average RMSD among the selected hits. This suggests a comparatively higher degree of backbone adjustment, while still maintaining an equilibrated trajectory during the simulation. Finally, the MMP2-S7397 complex displayed the lowest average RMSD (0.413 ± 0.066 nm), even lower than the apo form, co-crystallized ligand, and lead compound. This system showed initial fluctuations from 0.20 to 0.35 nm during the first 20 ns, stabilized at 0.35 nm until 50 ns, then fluctuated between 0.40 and 0.50 nm up to 125 ns, with a transient spike to 0.60 nm, before stabilizing again within 0.40–0.50 nm for the remainder of the simulation. Therefore, the RMSD data ranked S7397 as the most stable complex at the backbone level, whereas S1005 showed the largest deviation among the five selected hits.

RMSF was then used to compare residue-level mobility across the apo and ligand-bound systems. As illustrated in Figure 6d–f and Table 2, the RMSF values of the apo form, co-crystallized complex, lead compound, and the five docking-identified hits were compared. The plots revealed that residues within the ligand binding pocket exhibited lower fluctuations relative to other regions, suggesting that binding of the hit compounds stabilized the active site. The apo form of MMP2 displayed an average RMSF of 0.231 ± 0.134 nm, while the co-crystallized ligand MMP2-I52 and the lead compound MMP2-09 exhibited lower RMSF values of 0.166 ± 0.096 nm and 0.215 ± 0.111 nm, respectively, indicating that ligand binding reduced local flexibility compared to the unbound protein. Among the newly identified hits, the MMP2-S2788 complex displayed the lowest RMSF value among the five selected compounds (0.180 ± 0.130 nm), followed by MMP2-S2807 (0.184 ± 0.148 nm), MMP2-S1178 (0.191 ± 0.117 nm), and MMP2-S1005 (0.195 ± 0.127 nm). The MMP2-S7397 complex exhibited a slightly higher RMSF value of 0.218 ± 0.144 nm, which remained comparable to the lead compound and lower than the apo form. Thus, the RMSF profile did not follow the same order as RMSD: S7397 gave the lowest backbone deviation, whereas S2788 and S2807 showed the lowest average residue fluctuations. A residue-resolved inspection of the three flexible segments highlighted in Figure 6d–f further clarifies where this stabilization occurs. The region spanning residues 75–90 consistently emerged as the most mobile segment of MMP2, with the apo protein reaching an RMSF of approximately 0.70 nm most complexes reduced this peak substantially, with the co-crystallized ligand I52 showing the strongest reduction, whereas MMP2-S2807 remained comparably flexible to the apo form and MMP2-S7397 retained an elevated, flat profile across this stretch. In contrast, the 110–120 segment, which lies immediately adjacent to the zinc-coordinating His120, was markedly more rigid across all systems, with RMSF values generally falling below 0.10 nm by residue 120, the MMP2-09 was the only system to show a pronounced local peak within this segment, while the FDA-approved hits showed limited perturbation of this region of the catalytic pocket. The 130–150 segment displayed intermediate, ligand-dependent behavior, with the MMP2-09 and MMP2-S1005 showing the highest fluctuations among the evaluated systems, while the co-crystallized ligand I52, S2788, and S7397 remained comparatively stable. Taken together, these residue-level patterns indicate that the immediate catalytic environment of MMP2 was largely preserved across the selected hits, while the differences captured by the average RMSF values mainly reflect ligand-dependent stabilization, or lack thereof, of the flanking 75–90 and 130–150 loops rather than changes within the rigid 110–120 segment containing part of the active site.

Rg was calculated to examine whether ligand binding affected the compactness of MMP2 during the simulation. As shown in Figure 6g–i and Table 2, the Rg profiles were obtained for the apo form, co-crystallized complex MMP2-I52, lead compound MMP2-09, and the five selected hit complexes. The average Rg values were 1.539 ± 0.022 nm for apo MMP2, 1.530 ± 0.028 nm for MMP2-I52, and 1.544 ± 0.026 nm for MMP2-09. Among the selected hits, MMP2-S2807 showed the lowest Rg value (1.528 ± 0.024 nm), slightly lower than the apo, co-crystallized, and lead compound systems. MMP2-S1005 also showed a compact profile with an average Rg of 1.535 ± 0.021 nm, while MMP2-S7397 displayed a Rg value comparable to the apo protein (1.539 ± 0.026 nm). In contrast, MMP2-S2788 and MMP2-S1178 showed slightly higher Rg values of 1.555 ± 0.023 nm and 1.577 ± 0.032 nm, respectively, suggesting a modest increase in global protein expansion upon ligand binding. The Rg differences were small, but they separated the complexes from each other: S2807 and S1005 favored a more compact MMP2 conformation, whereas S1178 and S2788 produced slightly higher average Rg values. No marked expansion of the protein was observed in any trajectory.

SASA was finally analyzed to compare solvent exposure among the MMP2 systems. As shown in Figure 6j–l and Table 2, the average SASA values for the apo form, co-crystallized complex MMP2-I52, lead compound MMP2-09, and the selected hit complexes were 96.368 ± 3.229 nm^2^, 94.636 ± 3.201 nm^2^, 96.555 ± 2.546 nm^2^, 97.472 ± 2.744 nm^2^ for S1005, 96.856 ± 2.102 nm^2^ for S1178, 96.217 ± 2.376 nm^2^ for S2788, 94.569 ± 2.572 nm^2^ for S2807, and 99.059 ± 3.024 nm^2^ for S7397. Compared to the apo protein, the MMP2-I52 complex displayed a modest reduction in SASA, suggesting that the co-crystallized ligand stabilizes the protein structure by decreasing solvent exposure. Similarly, MMP2-S2807 showed the lowest SASA among the selected hits, lower than both the apo and lead complexes, suggesting that this compound contributes to a more compact and less solvent-exposed protein conformation. The MMP2-S2788 complex showed a SASA value close to that of the apo protein but slightly lower, indicating limited solvent exposure and stable protein packing. MMP2-S1005 and MMP2-S1178 showed slightly higher SASA values than the apo form, whereas MMP2-S7397 displayed the highest SASA value (99.059 ± 3.024 nm^2^). This may reflect increased solvent accessibility of surface residues, possibly linked to local conformational adjustments upon ligand binding. This result is important because S7397 had the lowest RMSD but the highest SASA, meaning that its favorable backbone stability was accompanied by greater solvent exposure. By contrast, S2807 combined low Rg and low SASA, while S2788 showed the lowest RMSF among the selected hits.

Hydrogen bond (HB) occupancy over the trajectory was also examined for the MMP2 complexes (Figure 6m–o) to complement the reported static interaction analysis. MMP2-S7397 maintained the most persistent hydrogen bonding network throughout the simulation, sustaining 3–4 hydrogen bonds for most of the 200 ns trajectory and transiently reaching 5–6, consistent with the seven hydrogen bonds identified in its initial docking pose and with its favorable RMSD profile. MMP2-S1178 maintained a comparatively stable hydrogen bond count of around 2–3 throughout the trajectory, in line with the hydrogen bonds and additional zinc-directed contacts observed at the docking stage. MMP2-S1005 showed a pronounced hydrogen bond enrichment during approximately the first 20 ns, transiently reaching 5–6, which then declined for the remainder of the simulation, indicating that its initial docking-predicted hydrogen bonding network was not fully sustained over the trajectory. MMP2-S2788 and MMP2-S2807 both maintained lower and broadly similar hydrogen bond counts, generally fluctuating between 1 and 2 throughout the simulation, consistent with the more hydrophobic-dominated binding modes described for these compounds. The shared reference traces, MMP2-I52 and MMP2-09, generally displayed low and intermittent hydrogen bond counts relative to S7397 and S1178, reflecting their comparatively lower reliance on sustained hydrogen bonding relative to zinc coordination and hydrophobic contacts. Overall, the persistence of hydrogen bonding over the trajectory highlighted S7397 and S1178 as the two hits showing the most sustained polar interactions among the five compounds. Therefore, the MMP2 MD analysis did not identify a single dominant compound across all metrics. Instead, it showed that the five MMP2-prioritized compounds remained relevant for different reasons and could be carried forward for secondary MMP3 profiling.

### 2.4. MM-PBSA Binding Free Energy Analysis of MMP2 Complexes

The MM-PBSA method was employed to estimate the binding free energies of the selected compounds toward MMP2 by decomposing the interaction into molecular mechanics (van der Waals and electrostatic) and solvation (polar PB and nonpolar surface) contributions. More negative ΔH_total_ values indicate stronger predicted ligand–protein association. The reported values represent averages over the sampled MD trajectory snapshots and are summarized in Table 3. Among the evaluated systems, I52 exhibited the most favorable binding free energy (ΔH_total_ = −31.24 ± 0.14), followed by S7397 (−30.09 ± 0.16), both outperforming the lead compound complex (3i, −29.29 ± 0.13). For these ligands, favorable binding is primarily driven by a strong van der Waals contribution together with a favorable gas-phase electrostatic term that is partly offset by the polar solvation penalty, consistent with typical MM-PBSA behavior. S1178 (−28.81 ± 0.12) showed a binding free energy comparable to the lead compound, whereas S2788 (−19.68 ± 0.10) and S1005 (−19.49 ± 0.19) formed a lower tier of moderately favorable complexes, and S2807 (−6.37 ± 0.20) displayed a markedly less favorable energetic profile. The MM-PBSA ranking aligns partially with the docking and MD results. S1178 and S7397, which ranked first and second by docking score, and it also produced the most favorable MM-PBSA energies among the five FDA-approved hits; both showed comparatively low RMSD in the MD simulations, providing convergent evidence that these two compounds are the most robust MMP2 binders identified in this study. Conversely, the co-crystallized ligand I52 obtained the least favorable docking score (−8.3 kcal/mol) yet the most favorable MM-PBSA energy, a discrepancy that likely reflects the limitations of empirical docking scoring functions in capturing the full energetics of a native, well-adapted binding pose. S2807 illustrates the converse pattern: despite a competitive docking score (−9.4 kcal/mol, tied with S1005) and the most compact Rg among the hits, it showed by far the smallest ΔE_gas_ magnitude and the least favorable ΔH_total_, indicating that its docking-predicted affinity was not sustained over the simulated trajectory and that this compound should be regarded as the least promising of the five candidates. S1005 similarly combined a modest MM-PBSA energy with the highest average RMSD among the hits, consistent with comparatively weaker overall stability. S2788, in contrast, showed the lowest RMSF (indicating a rigid local binding pocket) but only a moderate ΔH_total_, suggesting that local active site rigidity did not fully translate into a proportionally favorable net binding free energy, likely owing to its relatively large polar desolvation penalty. Taken together, the docking, MD, and MM-PBSA analyses converge in supporting S1178 and S7397 as the most promising repurposing candidates against MMP2, while highlighting S2807 as a case where the docking score alone would have overestimated binding affinity relative to the more rigorous MM-PBSA and MD-derived evidence.

### 2.5. Secondary Docking Profile of the Selected Compounds Against MMP3

**Figure 7:**
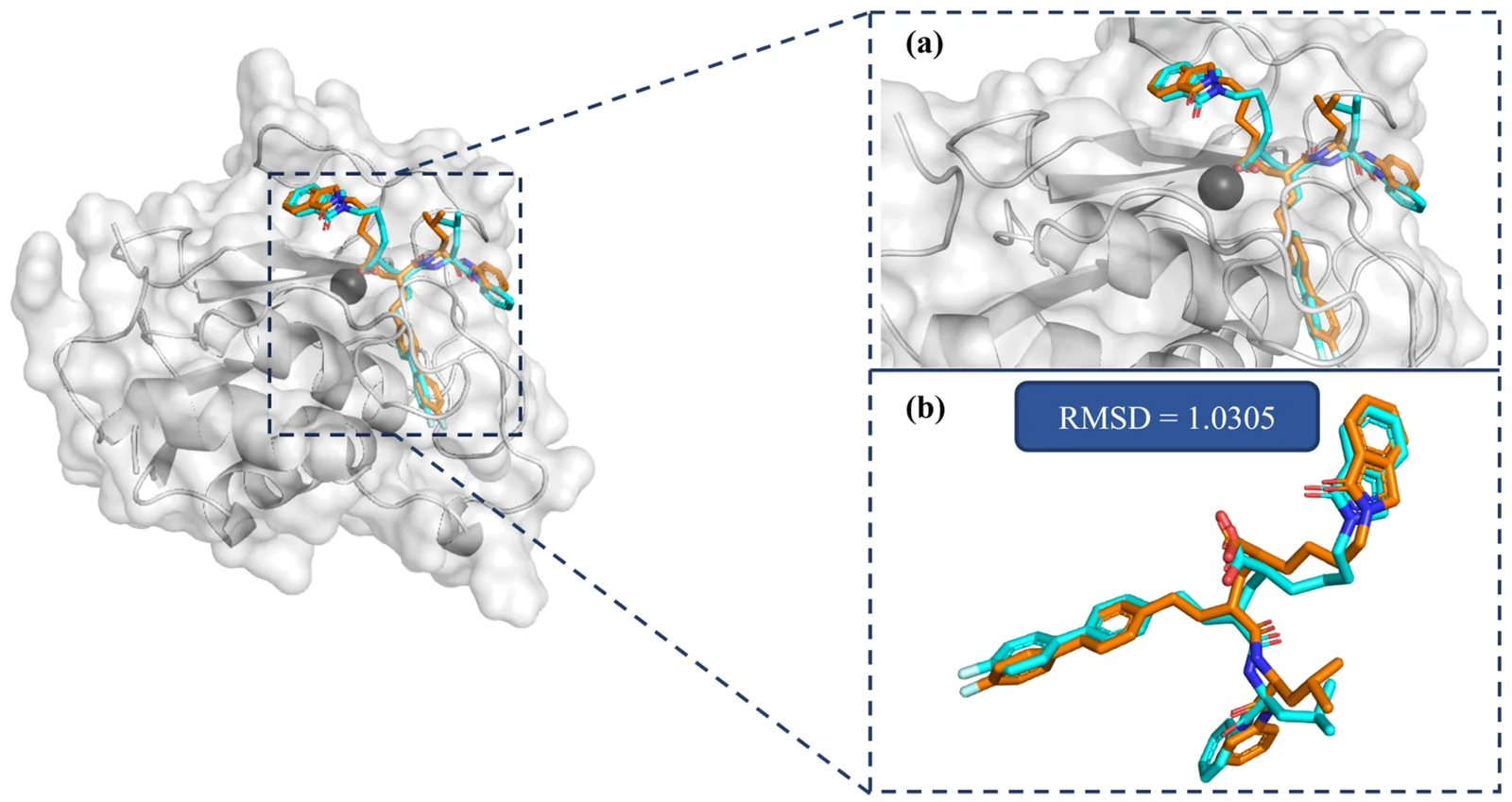
The docking protocol validation (**a**) zoomed view of the native ligand (orange) and docked pose (cyan) in the MMP3 active site. (**b**) Superposition of both conformations.

Following the MMP2 docking and MD analyses, the five compounds prioritized from the MMP2 workflow were further evaluated against MMP3 to examine whether any of them could retain favorable binding toward a related profibrotic metalloproteinase. Before docking these compounds, the MMP3 docking protocol was first validated. The 1HFS co-crystallized ligand (L04) was re-docked into the active site of MMP3. In general, a docking protocol is validated when the RMSD value is less than 2 Å. The results confirm the validation of the docking protocol with an RMSD value of 1.0305 Å. Figure 7 illustrates the superposition of the native and the re-docked ligand conformations, demonstrating the docking protocol’s reliability.

To investigate the potential of the five selected compounds beyond MMP2, a secondary docking study against MMP3 was subsequently conducted. The five MMP2-prioritized compounds—S1005, S1178, S2788, S2807, and S7397—were docked into the catalytic site of MMP3 and compared with the reference compound (Ref, orb1709104), lead compound (09) and the co-crystallized ligand L04. The reference compound was chosen based on its high inhibition activity (1 nM) [24]. Among the five compounds, S2788 showed the highest binding affinity (−11.5 kcal/mol), followed by S1178 (−11.4 kcal/mol), S7397 (−11.0 kcal/mol), S1005 (−10.9 kcal/mol), and S2807 (−9.4 kcal/mol). S2788 and S1178 were therefore the only compounds with docking scores better than L04 (−11.4 kcal/mol), Ref and 09 (−10.6 and −9.7 kcal/mol, respectively). Based on this ranking, S2788 and S1178 were selected for interaction analysis and subsequent MMP3 MD simulations, while the remaining three compounds were not advanced further in the MMP3 workflow. The docking scores and structures are summarized in Table 4 and Table S5.

**Figure 8:**
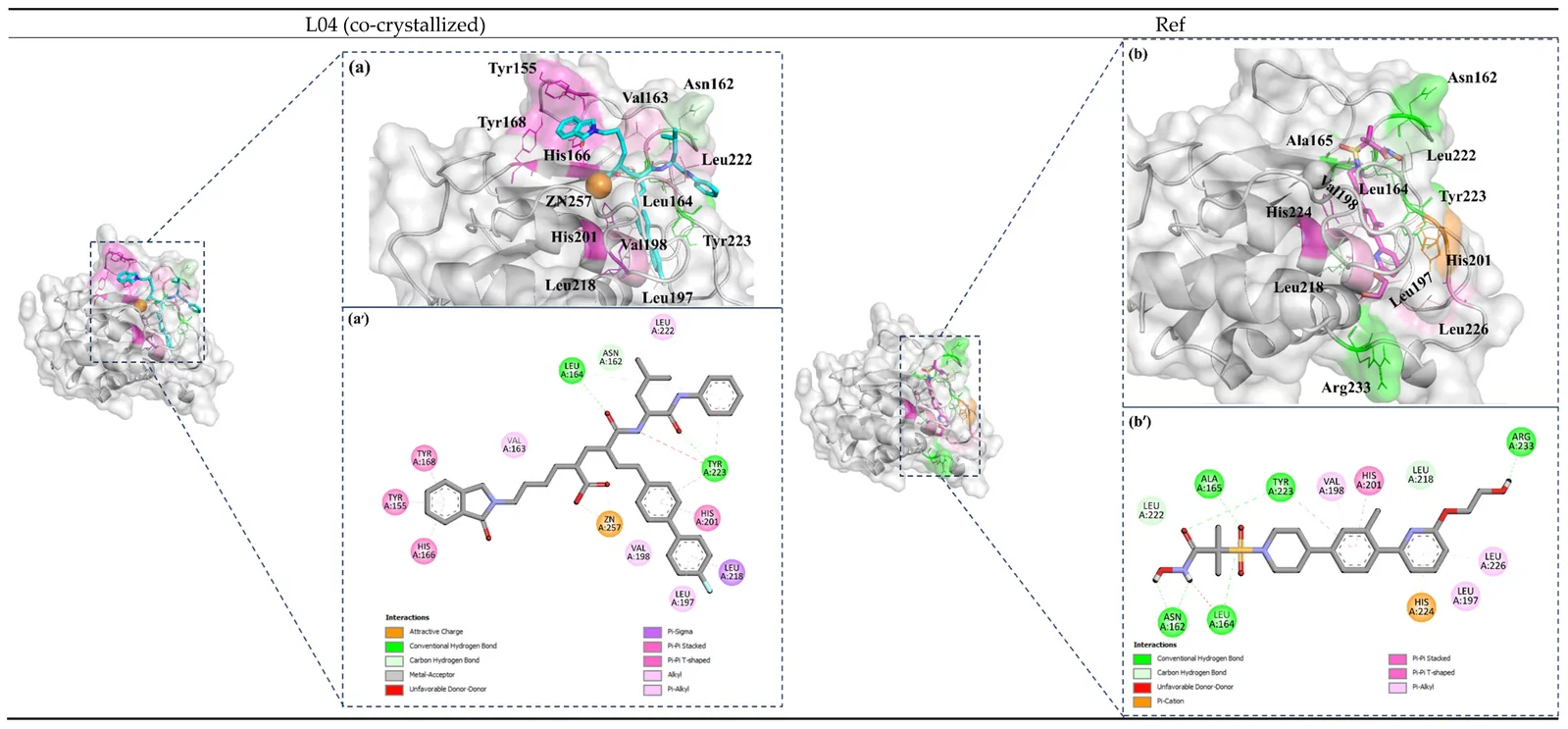

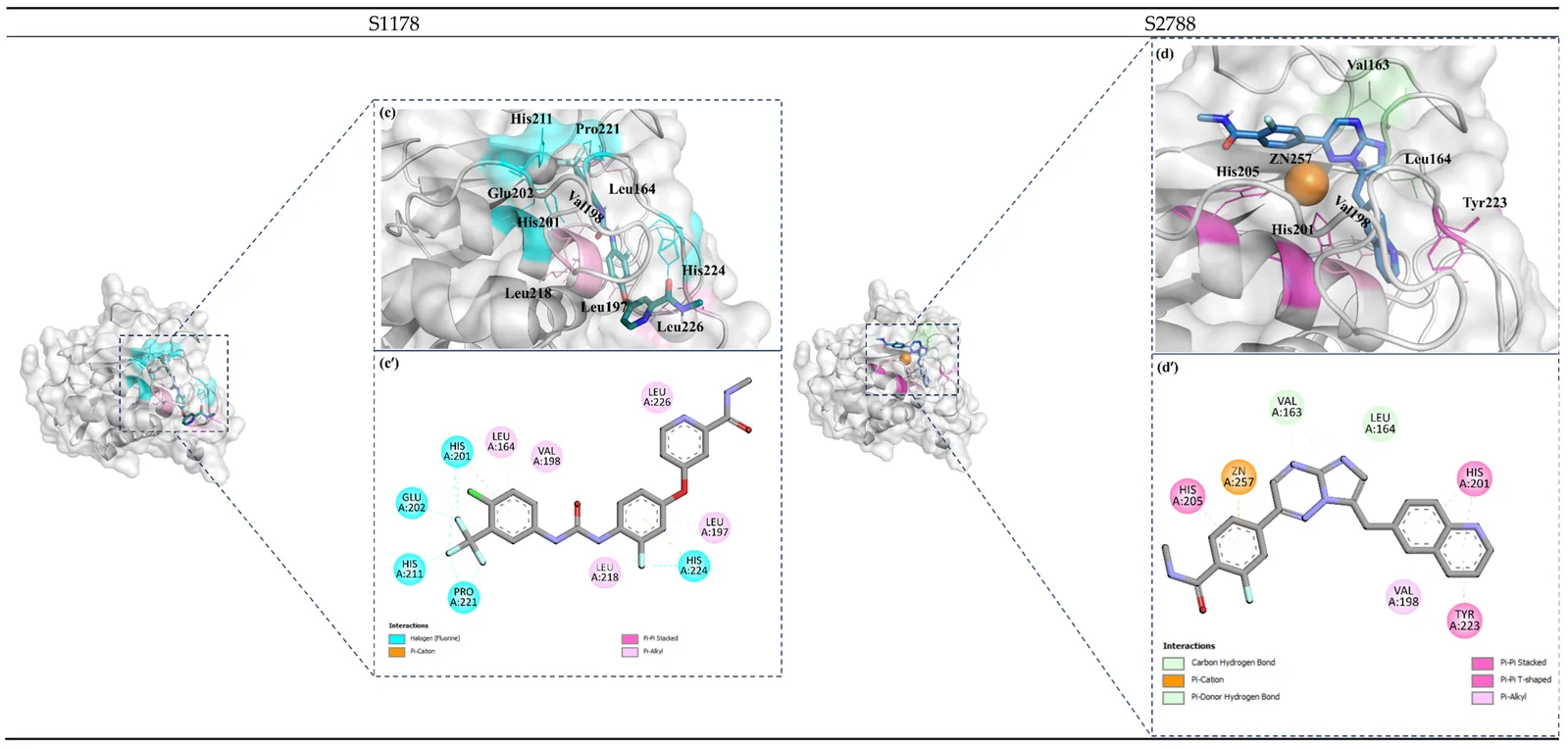
(**a**–**d**) 2D and (**a’**–**d’**) 3D interaction mapping of the two selected, reference and co-crystallized compounds within the active site of MMP3.

To examine the interaction profile of the two best-ranked MMP3 hits within the active site of MMP3, both two- and three-dimensional interaction mapping analyses were conducted (Figure 8). The interaction study indicated that the ref compound had a binding affinity of −10.6 kcal/mol and exhibited a strong binding profile within the pocket site of MMP3. It formed hydrogen bonds with Leu164, Ala165, Tyr223, Arg233, and Asn162, with distances ranging from 1.83 to 2.82 Å. It also showed an electrostatic π-cation interaction with His224. Multiple hydrophobic, π-π, and π-alkyl interactions with residues such as His201, His224, Tyr223, Leu197, Leu226, and Val198 stabilized the ref ligand within the active site of MMP3. In contrast, the co-crystallized ligand L04 showed a metal–acceptor interaction with Zn257 and hydrogen bonding interactions with Leu164, Tyr223, and Asn162. Multiple aromatic and additional hydrophobic interactions with residues such as His201, His166, Tyr223, Val163, and Val198 further supported its experimental activity.

In comparison to these references, the two selected FDA-approved drugs exhibited interaction patterns that both matched and enhanced some binding features of the ref and co-crystallized ligands. S1178 formed multiple halogen interactions involving the fluorine atom of the ligand with residues such as His201, Glu202, His211, Pro221, and His224. Additionally, S1178 formed π-alkyl hydrophobic interactions with Leu226, Leu197, and Val198. It also formed π-π stacking interactions with His201 and His224. Although S1178 did not reproduce the zinc coordination and hydrogen bonding pattern observed for L04, it occupied the same catalytic region through a binding mode dominated by halogen, hydrophobic, and aromatic contacts. Thus, S1178 appears to adopt a non-classical MMP3 binding mode, mainly stabilized by secondary noncovalent contacts rather than direct zinc coordination.

In contrast, S2788 displayed a more classical MMP3 binding profile. It was the only compound that showed interaction with Zn257 through a π-cation electrostatic interaction, similar to the L04 molecule. S2788 also formed hydrogen bond interactions with Val163 and Leu164. Moreover, S2788 formed multiple hydrophobic interactions with His201 and Val198, which mimicked the binding behavior of L04 and may explain its favorable binding affinity. Table S5 presents the detailed docking interactions of the five selected compounds, along with the ref and L04 compounds.

Out of the five FDA-approved compounds that emerged from the virtual screening campaign targeting MMP2, only two, S1178 and S2788, maintained a consistently strong and favorable binding profile when evaluated against MMP3 as a second target. Their selection can be explained by both their docking scores and the nature of their interactions within the MMP3 active site.

From a structural perspective, MMP2 and MMP3 are closely related members of the matrix metalloproteinase family. They both have a catalytic domain organized around a zinc-binding histidine triad, His120, His124, and His130 in MMP2, and His201, His205, and His211 in MMP3, along with a hydrophobic S1′ subpocket. In MMP2, residues such as Leu83 contribute to the binding region, whereas in MMP3 residues including Leu197 and Val198 participate in the hydrophobic pocket. Because of this shared catalytic architecture, ligands with suitable geometric and physicochemical properties may retain binding ability across both enzymes. However, this cross-target recognition does not necessarily occur through the same interaction pattern.

Regarding S1178, its performance across both proteins was notably consistent: it ranked as the top-scoring hit against MMP2 with a binding affinity of −10.1 kcal/mol and retained a highly competitive value of −11.4 kcal/mol against MMP3. What distinguishes S1178 from the other screened compounds is its reliance on a rich network of halogen-bonding interactions, rather than the classical zinc-directed binding pattern. In MMP2, S1178 interacted with residues such as Pro134, Gly135, and Ala136. In MMP3, it formed contacts with His201, Glu202, His211, Pro221, and His224. Furthermore, the compound engaged in π-π stacking contacts with histidine residues in the MMP3 active site, supporting its stabilization within the catalytic pocket. It is worth noting that S1178 does not appear to coordinate directly with the catalytic zinc ion in MMP3, unlike what is typically expected for classical high-affinity MMP binders. Instead, its docking profile suggests a non-classical binding mode in which halogen, hydrophobic, and aromatic contacts compensate for the absence of direct zinc coordination.

S2788, on the other hand, follows a more orthodox inhibition pattern that more closely mirrors the behavior of known MMP inhibitors and crystallographic ligands. In both enzyme systems, this compound preserved interaction with the catalytic region via a metal–acceptor contact in MMP2 and a π-cation electrostatic interaction with Zn257 in MMP3. Beyond zinc-directed recognition, S2788 reproduced several key contacts in the active sites, including Leu83 and His120 in MMP2, and Val163 and Leu164 in MMP3. It also simultaneously engaged the hydrophobic lining of the S1′ pocket. This interaction pattern explains why S2788 showed the strongest MMP3 docking score among the five tested compounds.

### 2.6. Molecular Dynamics Analysis of Selected MMP3 Complexes

**Figure 9:**
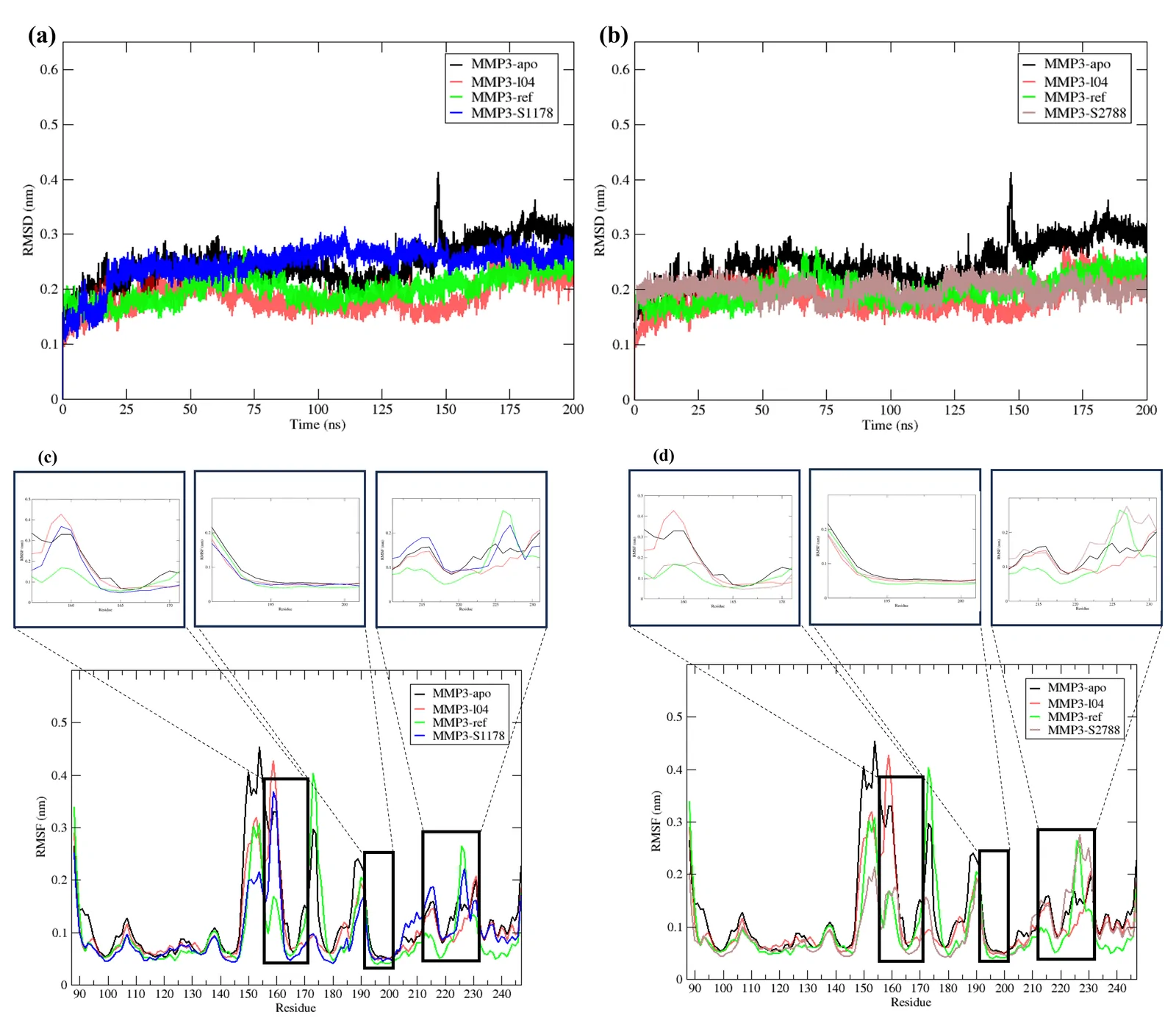

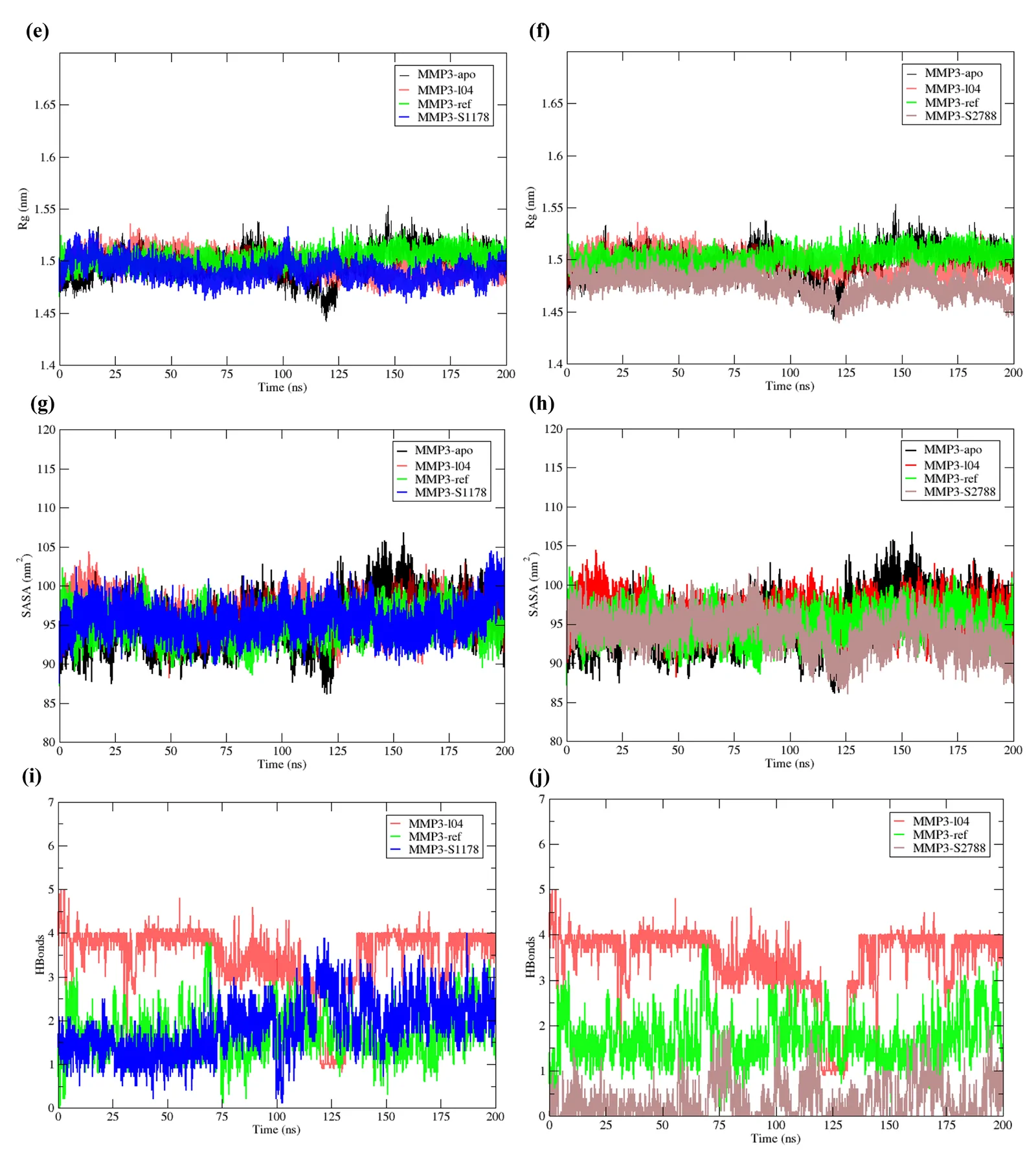
RMSD (**a**,**b**), RMSF (**c**,**d**), Rg (**e**,**f**), SASA (**g**,**h**) and HB (**i**,**j**) plots of co-crystallized ligand L04, reference compound, S1178, and S2788.

Based on the docking results of MMP3, only S1178 and S2788 were selected for MD simulations, since these two compounds showed the most favorable docking scores and interaction profiles among the five MMP2-prioritized hits. MD simulations were performed to evaluate the stability of the interactions between MMP3 and the two selected ligands, as well as to estimate their binding behavior over time. The apo form of MMP3 (MMP3-apo), the MMP3 complexed with co-crystallized ligand (MMP3-L04), reference compound (MMP3-Ref) were used for comparison. Following the production runs, several structural and dynamic parameters—RMSD, RMSF, Rg, and SASA—were calculated to assess the protein–ligand stability (Table 5 and Figure 9).

Backbone RMSD was first examined to compare the global stability of the MMP3 systems during the 200 ns simulations. As shown in Figure 9a,b and Table 5, the RMSD of the apo form showed the highest structural fluctuation, with the RMSD increasing gradually from 0.20 nm to around 0.35 nm during the simulation, except for a noticeable spike near 150 ns, indicating larger conformational movement in the absence of a ligand. For the MMP3-L04 complex, the RMSD remained relatively low and stable during the simulation, ranging from 0.18 nm to around 0.22 nm, showing that the co-crystallized ligand L04 stabilized the protein structure effectively. For the MMP3-Ref complex, the RMSD was maintained between 0.18 nm and 0.25 nm over the 200 ns simulation, showing slightly higher fluctuation than the co-crystallized L04 complex but still indicating good structural stability. The MMP3-S1178 complex exhibited RMSD values between 0.15 nm and 0.22 nm during the first 30 ns, then stabilized around 0.22–0.30 nm for the last 170 ns, indicating that the complex system remained stable during the simulation, although with slightly higher deviation than the co-crystallized ligand complex. The MMP3-S2788 complex exhibited RMSD values close to those of the co-crystallized ligand and lower than the apo form, remaining around 0.18–0.24 nm with limited fluctuation during the simulation. Thus, the RMSD profile separated the two compounds: S1178 remained stable but close to the apo system, whereas S2788 behaved closer to the ref and co-crystallized systems.

RMSF was then analyzed to compare residue-level flexibility among the MMP3 systems. As illustrated in Figure 9c,d and Table 5, the apo form of MMP3 displayed an RMSF average value of 0.125 ± 0.083 nm, representing the highest fluctuation among the analyzed systems, particularly around residues 145–165 and 175–185. This indicates greater flexibility in these regions in the absence of a ligand. For the MMP3-L04 complex, the RMSF average value was 0.106 ± 0.068 nm, reducing the magnitude of these fluctuations in the presence of the co-crystallized ligand. For the MMP3-Ref complex, the RMSF average value was 0.096 ± 0.068 nm, showing a similar pattern to the L04 complex but with a slightly lower average fluctuation. The MMP3-S1178 complex had an RMSF average value of 0.097 ± 0.057 nm, comparable to the ref ligand. Figure 9c,d also demonstrates relatively lower RMSF values across most residues, indicating that S1178 contributes to stabilizing several regions of the protein, especially the active site region. The MMP3-S2788 complex presented RMSF values lower than the co-crystallized ligand, with an average value of 0.093 ± 0.051 nm, indicating that S2788 contributed to the stability of the protein backbone. The RMSF data therefore favored S2788, followed closely by S1178 and the ref complex. A residue-resolved inspection of the three flexible segments highlighted in Figure 9c,d further clarifies where this stabilization occurs for MMP3. The region spanning residues 156–171 was the most mobile segment in this range, with the apo protein and the co-crystallized ligand L04 reaching the highest RMSF values (peaking at approximately 0.33 and 0.42 nm, respectively) the reference compound produced the strongest reduction in fluctuation of this loop, remaining below 0.17 nm throughout, while MMP3-S1178 tracked closely with the apo protein and offered only limited stabilization, and MMP3-S2788 remained comparably low to the reference compound. In contrast, the 191–201 segment behaved similarly across all systems, with RMSF values declining steadily from approximately 0.20 nm to below 0.10 nm irrespective of ligand identity, indicating that this stretch, which lies close to the zinc-binding histidine triad (His201, His205, His211), is intrinsically rigid and not differentially modulated by ligand binding. The 211–231 segment displayed the most ligand-dependent behavior among the three: in the MMP3-S1178 system, this region showed a localized rise in flexibility (up to approximately 0.22 nm) not observed for the co-crystallized ligand or the reference compound, whereas in the MMP3-S2788 system the reference compound itself displayed a pronounced, isolated spike (approaching 0.4 nm) that was not reproduced by S2788, which instead remained comparatively low across this segment. Taken together, these residue-level patterns show that, as in MMP2, the immediate catalytic environment of MMP3 remained largely rigid across all evaluated systems, while the differences in average RMSF were mainly driven by variable stabilization of the flanking 156–171 and 211–231 loops rather than by changes at the catalytic core itself.

Rg was calculated to assess whether S1178 and S2788 affected the compactness of MMP3. Figure 9e,f and Table 5 illustrate the Rg profile for each system. The apo form had an average value of 1.498 ± 0.014 nm, with higher fluctuation especially around 100–120 ns, suggesting a less compact apo state during this period. The MMP3-L04 complex had an average Rg value of 1.497 ± 0.010 nm and remained stable during the simulation. The MMP3-Ref complex had an average value of 1.502 ± 0.008 nm, which was slightly higher than that of the co-crystallized complex. For the MMP3-S1178 complex, the Rg value was 1.491 ± 0.010 nm, representing the second-lowest value among the systems and showing a stable profile with small fluctuations. The MMP3-S2788 complex showed the lowest Rg value of 1.476 ± 0.010 nm, meaning that the protein became slightly more compact in the presence of this ligand. Compared with apo MMP3 and the ref systems, S1178 and especially S2788 favored a more compact MMP3 conformation.

SASA was finally used to compare solvent exposure to the MMP3 systems. Figure 9g,h presents the SASA profiles for the apo form, co-crystallized ligand L04, ref compound, S1178, and S2788, with average SASA values of 95.456 ± 3.003 nm^2^, 96.249 ± 1.944 nm^2^, 95.066 ± 1.842 nm^2^, 95.558 ± 1.964 nm^2^, and 93.854 ± 2.261 nm^2^, respectively. As shown in Figure 9g,h and Table 5, the average SASA value for the apo form was 95.456 ± 3.003 nm^2^. However, the co-crystallized MMP3-L04 complex exhibited a slightly larger average value of 96.249 ± 1.944 nm^2^, suggesting that more protein residues were exposed to the solvent. The ref complex exhibited a similar average SASA value of 95.066 ± 1.842 nm^2^ with a slight decrease, indicating that the ref ligand reduced solvent exposure. For the chosen compounds, the MMP3-S1178 complex exhibited an average SASA value of 95.558 ± 1.964 nm^2^, comparable to the apo form, indicating minimal structural perturbation. In contrast, the MMP3-S2788 complex displayed a lower SASA value of 93.854 ± 2.261 nm^2^, which may reflect reduced solvent accessibility of surface residues, possibly associated with increased compactness upon ligand binding. This agrees with the Rg profile, where S2788 also showed the most compact MMP3 conformation.

Hydrogen bond counts were similarly monitored for the MMP3 systems (Figure 9i,j). The co-crystallized ligand MMP3-L04 maintained the most extensive and persistent hydrogen bonding network among all systems, sustaining 4–5 hydrogen bonds for most of the trajectory and transiently reaching 6 HBs, consistent with its role as a native ligand. The Ref compound maintained a comparatively lower and more variable hydrogen bond count, generally fluctuating between 2 and 3 HBs. MMP3-S1178 sustained a hydrogen bond count comparable to the Ref compound (around 3–4) during approximately the first half of the simulation, but this count declined toward 0–2 hydrogen bonds after approximately 110 ns, indicating a partial loss of direct hydrogen bonding contacts in the later stages of the trajectory. This is consistent with the non-classical, halogen- and hydrophobic-dominated binding mode proposed for S1178 rather than a hydrogen bond-driven mechanism. MMP3-S2788 maintained a lower and comparatively stable hydrogen bond count throughout the simulation, generally fluctuating between 1 and 2, consistent with the hydrogen bonds identified for this compound in the initial MMP3 docking analysis. Taken together, the hydrogen bond persistence data indicate that L04 relies on an extensive and sustained hydrogen bonding network, whereas S1178 and S2788 achieve MMP3 binding through comparatively fewer, and in the case of S1178, less consistently maintained hydrogen bonds, complementing the RMSD, RMSF, Rg, and SASA-based stability picture already discussed above.

Overall, the MMP3 MD analysis showed that both selected compounds remained stable during the 200 ns simulations, although their profiles were not identical. S1178 showed acceptable stability and maintained residue-level fluctuations comparable to the ref system. S2788 showed numerically more favorable values, with RMSD close to the ref complex, the lowest RMSF, the lowest Rg, and the lowest SASA. However, as each system was simulated in a single 200 ns trajectory and several of these differences are modest relative to the within-trajectory fluctuations, this comparison should be regarded as indicative rather than definitive evidence that S2788 forms a more stable MMP3 complex than S1178, independent replicate simulations would be required to confirm this ranking. Both compounds remained relevant candidates, with S1178 additionally supported by its favorable docking score and persistent noncovalent interaction profile.

### 2.7. MM-PBSA Binding Free Energy Analysis of MMP3 Complexes

The MM-PBSA approach was employed to estimate the binding free energies of the compounds evaluated against MMP3, including the co-crystallized ligand L04, the reference compound, and the two candidates selected from the MMP3 docking analysis, S1178 and S2788. The calculations were performed using the same energy decomposition strategy previously applied to MMP2. The resulting binding energy components, summarized in Table 6, represent average values derived from the same 200 ns MD trajectories described previously. Among the evaluated systems, the co-crystallized ligand L04 displayed the most favorable binding free energy (ΔH_total_ = −32.28 ± 0.14 kcal/mol), closely matched by S2788 (−31.61 ± 0.14 kcal/mol). For both complexes, this favorable energetics is dominated by a strong van der Waals contribution (ΔE_vdw_ = −57.51 and −59.97 kcal/mol, respectively) combined with a comparatively modest electrostatic term, consistent with the classical, hydrophobic, and zinc-anchored binding mode described for these two ligands. S1178 showed a substantially less favorable ΔH_total_ (−16.70 ± 0.13 kcal/mol), roughly half the magnitude obtained for L04 and S2788, reflecting a smaller van der Waals contribution (−32.12 kcal/mol) and its comparatively more limited hydrogen bonding network.

This MM-PBSA ranking is consistent with the MMP3 docking and MD results discussed above. S2788, which obtained the strongest docking score among the MMP3 hits together with the lowest RMSD, RMSF, Rg and SASA values recorded for the MMP3 complexes, produced an MM-PBSA energy nearly equal to that of the co-crystallized ligand, providing convergent evidence that it forms the more robust secondary interaction with MMP3. S1178, despite its favorable docking score and stable non-classical, histidine-directed binding mode, yielded a markedly lower MM-PBSA energy, mirroring the partial loss of hydrogen bonding contacts observed after approximately 110 ns in its MD trajectory. Taken together, the docking, MD and MM-PBSA analyses converge in supporting S2788 as the more energetically favorable of the two MMP3 candidates, while S1178 remains a plausible but comparatively weaker secondary hit whose MMP3 activity should be interpreted with additional caution.

## 3. Discussion

The present workflow identified five FDA-approved drugs with favorable MMP2-oriented pharmacophore and docking profiles, which were subsequently evaluated against MMP3. This secondary profiling highlighted Regorafenib (S1178) and Capmatinib (S2788) as the most promising candidates for dual-target consideration, supporting their prioritization for further validation. A key consideration is selectivity within the MMP family. Because the catalytic zinc-binding architecture is highly conserved, compounds interacting with this region may also recognize other MMPs. However, selectivity can be influenced by differences in the geometry and physicochemical properties of surrounding substrate-recognition regions [25,26,27]. In the present docking analyses, the prioritized compounds also formed hydrophobic, polar, and residue-specific interactions outside the immediate zinc-binding environment, which may contribute to differential recognition of MMP2 and MMP3. Nevertheless, MMP2/MMP3 selectivity cannot be established from the present data alone, and comparative profiling against additional MMPs and related metalloproteinases will be required. The predicted activity of MMP2-prioritized compounds toward MMP3 suggests that dual MMP2/MMP3 modulation may affect complementary components of fibrotic remodeling. Although this provides a mechanistic rationale for pursuing dual-active compounds, the therapeutic advantage of simultaneous inhibition over single-target modulation remains to be established experimentally. Comparative biochemical and functional studies will therefore be necessary to determine whether dual targeting improves antifibrotic activity without increasing undesirable off-target effects. The five prioritized compounds are clinically characterized as FDA-approved kinase inhibitors. Their principal targets, approved indications, major safety considerations, and relevant pharmacokinetic properties are summarized in Table 7. Their established clinical profiles are advantageous for repurposing because they provide existing information on systemic exposure, tolerability, and dose-related toxicities. Among the final candidates, Regorafenib and Capmatinib showed favorable and complementary computational profiles across MMP2 and MMP3. However, clinically achievable plasma concentrations cannot yet be translated into expected MMP2/MMP3 inhibition because experimental binding and inhibitory constants have not been determined. Lung tissue exposure also remains unknown. Safety considerations are particularly relevant for chronic treatment of IPF. Regorafenib is associated with hepatotoxicity, whereas Capmatinib carries a risk of ILD/pneumonitis in addition to hepatotoxicity [28,29,30]. These established safety profiles do not preclude repurposing but indicate that dose optimization and benefit–risk assessment will be essential if MMP2/MMP3 inhibition is confirmed experimentally. Taken together, the present study narrows a large FDA-approved drug library to a small set of clinically characterized candidates with predicted MMP2/MMP3 activity and favorable dynamic behavior. The findings provide a focused basis for subsequent experimental validation, including direct determination of MMP2 and MMP3 inhibitory potency, broader metalloproteinase selectivity profiling, and evaluation of pharmacologically relevant exposure and antifibrotic activity.

## 4. Materials and Methods

### 4.1. Data Collection and Preparation

#### 4.1.1. Sulfonamide Derivative Dataset Preparation

A dataset of 25 sulfonamide derivatives with their corresponding MMP2 inhibitory activities was collected from the research paper of Yan et al. (Table S1) [36]. The 2D chemical structures were drawn using ChemDraw (v23.1.2.7) [37], and their geometries were energy-minimized using the Merck Molecular Force Field 94 (MMFF94) in Chem3D. The half maximal inhibitory concentration (IC_50_) values were converted into their corresponding pIC_50_ values using the equation ${pIC}_{50}=-{log}_{10}({IC}_{50})$. The optimized structures, together with their pIC_50_ values, were used as the working dataset for pharmacophore modeling.

#### 4.1.2. Active/Inactive Generation

To validate the pharmacophore model and molecular docking protocol, a decoy (inactive) dataset was generated using A Database of Useful Decoys-Enhanced (DUD-E) database [38]. For each active compound, 50 inactives were generated. These inactive compounds were designed to have similar physicochemical properties to active compounds but different 2D topologies. The active/inactive dataset contained 1275 molecules (25 actives + 1250 inactives) and it was used to evaluate the reliability of the VS workflow.

#### 4.1.3. FDA-Approved Drug Library Preparation

The FDA-approved drug library was downloaded from SelleckChem [39]. The original file contained 3197 compounds. Before VS, the database was curated by correcting valence issues, standardizing functional-group and aromaticity representations, and removing duplicate entries. Following this process, 3020 unique compound structures were retained for virtual screening. The compound identifiers and Simplified Molecular Input Line Entry System (SMILES) strings are provided in the Supplementary Excel file.

### 4.2. Pharmacophore Model Generation and Validation

Ligand-based pharmacophore models were generated using LigandScout version 4.5 [40]. The active compounds were imported into the software, and conformers were generated using the iCon Best algorithm with default parameters. The full sulfonamide dataset comprised 25 compounds. The dataset was randomly divided into training and test sets at a 70:30 ratio, comprising approximately 18 and 7 compounds, respectively. Pharmacophore features were extracted from the aligned conformations, and 10 pharmacophore models were generated. The best model was selected based on the alignment score, which reflects the overlap of key chemical features among the active compounds [41]. The selected pharmacophore model was validated using the active/decoy dataset described above. Its ability to distinguish active compounds from inactive molecules was evaluated using several statistical parameters. Sensitivity, also known as the true positive rate (TPR), measures the ability of the model to correctly identify active compounds, whereas Specificity, also known as the true negative rate (TNR) [42], reflects its capacity to exclude inactive molecules. Accuracy (ACC) was used to evaluate the overall proportion of correctly classified active and inactive compounds. The ROC curve was used to provide a graphical representation of model performance by plotting the TPR against the false positive rate (FPR=1−Specificity). The AUC quantifies the overall discriminatory ability of the model, with values closer to 1 indicating stronger separation between active and inactive compounds [43]. In addition, *EF* was calculated to assess the early enrichment of active compounds among the retrieved hits [44], while *GH* was used as an overall quality metric that considers both the number of active hits and the total number of retrieved compounds [45]. These parameters were calculated using the following equations:

(1) $$Sensitivity\left(TPR\right)=\frac{TP}{TP+FN}=\frac{TP}{A}$$

(2) $$Specificity\left(TNR\right)=\frac{TN}{TN+FP}=\frac{TN}{D}$$

(3) $$ACC=\frac{TP+TN}{A+D}$$

(4) $$GH=\left(\frac{Ha\left(3A+Ht\right)}{4HtA}\right)\times \left(1-\frac{\left(Ht-Ha\right)}{\left(T-A\right)}\right)$$

(5) $${EF}_{x\%}=\frac{\frac{y_{a}}{x\%\;of\;D}}{\frac{TP\;+\;FN}{D}}$$

where *y_a_* is the number of actives retrieved within the top-ranked fraction of the dataset, *TP* represents true positives, *FN* represents false negatives, *TN* represents true negatives, *FP* represents false positives, *A* is the total number of active compounds, *D* is the total number of inactive compounds, and *T* is the total number of compounds in the validation dataset. *Ha* represents the number of active compounds retrieved as hits, *Ht* is the total number of hits retrieved.

### 4.3. Pharmacophore-Based Virtual Screening

The validated pharmacophore model was used to screen the curated FDA-approved drug library using LigandScout version 4.5. Compounds matching at least 70% of the 10 pharmacophoric features were retained as hits. This threshold was applied to increase screening flexibility and scaffold diversity. The selected hits were then subjected to molecular docking to evaluate their binding affinity and interaction profiles against MMP2.

### 4.4. Molecular Docking

Molecular docking was used to investigate the binding affinities and ligand–protein interactions of the FDA drugs screened by the pharmacophore model. The 3D structures of MMP2 (PDB ID: 1HOV) [46] and the MMP3 (PDB ID: 1HFS) [47] were retrieved from the RCSB Protein Data Bank. Water molecules and co-crystallized ligands were removed, while the catalytic zinc ion was retained. The protonation states of the protein structures were then assigned using the H++ web server [48]. For the ligands, no adjustments were performed, and they were used as provided in the FDA-approved library. Kollman charges were added using AutoDockTools v1.5.7 [49], and the proteins were saved in PDBQT format. The ligand structures were also converted and saved in PDBQT format using the same software. The grid boxes of MMP2 and MMP3 were defined based on their corresponding co-crystalized ligands (I52 and L04). The grid-box were set at (x = 5.67, y = 18.80, z = 22.39) with box size of 25 Å and (x = 35.34, y = 36.47, z = 24.17) with box size of 28 Å for MMP2 and MMP3, respectively. For both targets, a grid spacing of 0.375 Å was used to define the docking search space. The Ligand structures were similarly converted to PDBQT format using the same software. Molecular docking simulations were performed using AutoDock Vina software v1.1.2 [50] with an exhaustiveness value of 20 and the number of binding modes fixed at the default value of 9. The docking results were analyzed and visualized using Discovery Studio Visualizer (v25.1.0.24284) and PyMOL software (v3.1.5.1, edu, 2025) [51,52]. The reliability of the docking protocol was validated by re-docking the co-crystallized ligands (I52 and L04) into the binding pocket of MMP2 and MMP3, respectively, followed by calculation of RMSD. In this context, RMSD quantifies the positional deviation between the re-docked ligand pose and the experimentally determined native pose in the crystal structure, with lower values indicating a closer reproduction of the experimental binding mode. Additionally, the docking protocol was evaluated for its ability to distinguish between active and decoy compounds. The active/decoy dataset described earlier was docked under the same conditions. The resulting data were analyzed to compute sensitivity, Specificity, and ROC curves. The AUC here was used to quantify the ability of the docking protocol to distinguish actives from decoys. High sensitivity, Specificity, and AUC values confirmed the reliability of the docking method for VS. These metrics were generated using Screening Explorer [53].

### 4.5. Molecular Dynamics Simulation

In this study, both proteins MMP2 and MMP3 in the apo form and complexed with the chosen ligands were subjected to all-atom MD simulation using GROMACS version 2025.2 [54]. Prior to the simulations, the proteins were parameterized using the CHARMM36 force field [55], while the ligands were parameterized with the CHARMM General Force Field (CGenFF) for small molecules [56]. Given the presence of a zinc ion in the catalytic domain of both proteins MMP2 and MMP3, special attention was paid to its parametrization. The zinc ion was modeled using parameters compatible with the CHARMM force field, its charge (+2) was assigned, and coordination between the Zn2+ and the surrounding residues, especially the three histidines in both cases, was preserved by maintaining the geometry obtained from the crystal structure, following a non-bonded model. In order to obtain a more realistic representation of the metal coordination without imposing constraints on the ligand binding [57]. Each protein–ligand complex was solvated in a cubic simulation box filled with TIP3P water molecules [58], and counterions (Na^+^, Cl^−^) were added to neutralize the system and maintain a physiological salt concentration of 0.15 M. Periodic boundary conditions (PBC) were applied to mimic an infinite system, with box size and shape optimized to avoid interactions between periodic images.

Non-bonded interactions were modeled using the Lennard-Jones and Coulombic potentials with a cut-off distance of 12 Å. The Verlet cut-off scheme was applied to update the neighbor search list efficiently. Long-range electrostatic interactions were treated using the Particle Mesh Ewald (PME) method [59]. Bond constraints were applied using the Linear Constraint Solver (LINCS) algorithm [60], with all covalent bonds, including those involving hydrogen atoms, constrained during the simulations. Energy minimization of each system was first performed using the steepest descent algorithm with a maximum of 50,000 steps and a convergence criterion of 10 kJ/mol for the maximum force. System equilibration was then carried out in a two-step process. First, the systems were equilibrated under a canonical NVT ensemble for 100 ps at 300 K. Subsequently, equilibration continued under an NPT ensemble for 100 ps at 300 K and 1 bar. The V-rescale thermostat [61] and the Berendsen barostat [62] were employed to regulate the temperature and pressure, respectively. Finally, a 200 ns production run was performed to evaluate the stability of the apo forms of MMP2 and MMP3, and compounds within their binding pockets. The structural stability of the protein–ligand complexes was assessed using RMSD, radius of gyration (Rg), root mean square fluctuation (RMSF), solvent accessible surface area (SASA), and HB based on the simulation trajectories. The resulting data were analyzed using XMGrace software version 5.1.22 [63].

### 4.6. MM-PBSA Binding Free Energy Analysis

The binding free energy was calculated using the MM-PBSA methods, which integrates molecular mechanics and continuum solvent models. The binding free energy (∆*G_binding_*) is calculated using the following equation:

(6) $$∆G_{binding}=∆G_{PL}-\left[∆G_{P}+∆G_{L}\right]$$

where *PL*, *P* and *L* represent the free energy of protein–ligand, protein and ligand, respectively; these energies are further estimated according to the following equation:

(7) $$∆G_{binding}=∆G_{gas}+∆G_{solv}=∆G_{vdw}+∆G_{ele}+∆G_{polar}+∆G_{nonpolar}$$

In this equation, Δ*E_gas_* corresponds to the gas-phase potential energy arising from van der Waals and electrostatic interactions, while Δ*G_solv_* represents for the solvation free energy, including polar and nonpolar components. MM-PBSA calculations were carried out using gmx_MMPBSA (v1.6.5 2026) [64]. The analysis was based on the final 100 ns of the MDs trajectory, from which 10,000 snapshots were uniformly extracted from 200,000 frames at an interval of 10. The results were subsequently visualized using the gmx_MMPBSA_ana tool (v1.6.5, 23 May 2026).

## 5. Conclusions

In this study, an integrated in silico workflow was applied to identify FDA-approved drugs as candidate MMP2 inhibitors with potential cross-activity against MMP3, two MMPs involved in extracellular matrix remodeling and IPF progression. A ligand-based pharmacophore model was first developed, and the selected model showed strong validation performance to support its use for virtual screening. Screening of the FDA-approved drug library yielded 83 pharmacophore-matching compounds, which were then prioritized through molecular docking against MMP2. Among the screened compounds, five molecules (S1005, S1178, S2788, S2807, and S7397) showed more favorable binding affinities with MMP2 than both the lead compound and the co-crystallized ligand. The MD results showed that the selected compounds maintained acceptable stability during the simulations. Because MMP3 is also implicated in profibrotic extracellular matrix remodeling, the same five MMP2-prioritized compounds were subsequently evaluated against MMP3 as a secondary cross-target profiling step. Among them, S2788 and S1178 showed the most favorable MMP3 docking profiles. S2788 displayed a more classical binding mode involving Zn257 and hydrogen bond interactions, whereas S1178 adopted a non-classical binding pattern mainly stabilized by halogen and hydrophobic interactions. MD simulations of these two MMP3 complexes further supported their stability, with S2788 showing the most consistent profile based on MD metrics values. Collectively, these findings suggest that S1178 and S2788 are promising MMP2-prioritized candidates with potential MMP3 cross-activity. However, this study remains computational and should be considered a prioritization step. Further experimental validation is required, including enzymatic assays against MMP2 and MMP3, studies on activated fibroblasts derived from IPF patients, and in vivo evaluation using a bleomycin-induced pulmonary fibrosis model. These future investigations will be essential to confirm the inhibitory activity, selectivity, and antifibrotic relevance of the proposed candidates.

## Figures and Tables

**Table 1:** Pharmacophore fit and docking scores of the selected FDA-approved compounds complexed with MMP2, along with the lead compound (09) and the co-crystallized ligand (I52).

Mol_ID	Name	Pharmacophore Fit	Docking Score (Kcal/mol)
S1178	Regorafenib	77.21	−10.1
S7397	Sorafenib	77.07	−10.0
S2788	Capmatinib	75.92	−9.8
S2807	Dabrafenib	76.79	−9.4
S1005	Axitinib	76.08	−9.4
09	4-(3-(2,4-dimethylphenyl)-5-(naphthalen-1-yl)-4,5-dihydro-1H-pyrazol-1-yl)benzenesulfonamide	107.69	−9.3
I52	N-{4-[(1-Hydroxycarbamoyl-2-Methyl-Propyl)-(2-Morpholin-4-Yl-Ethyl)-Sulfamoyl]-4-Pentyl-Benzamide	--	−8.3

**Table 2:** Calculated average parameters and over a 200 ns MD simulation for all systems of MMP2 along with 95% confidence intervals.

System	RMSD (nm)	RMSF (nm)	Rg (nm)	SASA (nm^2^)
MMP2-apo	0.444 ± 0.049 (0.444–0.445)	0.231 ± 0.134 (0.209–0.253)	1.539 ± 0.022 (1.539–1.539)	96.368 ± 3.229 (96.323–96.413)
MMP2-I52	0.462 ± 0.034 (0.462–0.463)	0.166 ± 0.096 (0.151–0.182)	1.530 ± 0.028 (1.529–1.530)	94.636 ± 3.201 (94.592–94.680)
MMP2-09	0.495 ± 0.061 (0.495–0.496)	0.215 ± 0.111 (0.197–0.233)	1.544 ± 0.026 (1.543–1.544)	96.555 ± 2.546 (96.520–96.590)
MMP2-S1005	0.572 ± 0.079 (0.571–0.574)	0.195 ± 0.127 (0.174–0.215)	1.535 ± 0.021 (1.535–1.536)	97.472 ± 2.744 (97.434–97.510)
MMP2-S1178	0.471 ± 0.086 (0.470–0.472)	0.191 ± 0.117 (0.172–0.210)	1.577 ± 0.032 (1.577–1.578)	96.856 ± 2.102 (96.827–96.885)
MMP2-S2788	0.520 ± 0.071 (0.519–0.521)	0.180 ± 0.130 (0.159–0.201)	1.555 ± 0.023 (1.555–1.555)	96.217 ± 2.376 (96.184–96.250)
MMP2-S2807	0.501 ± 0.047 (0.500–0.501)	0.184 ± 0.148 (0.160–0.208)	1.528 ± 0.024 (1.528–1.528)	94.569 ± 2.572 (94.533–94.605)
MMP2-S7397	0.413 ± 0.066 (0.412–0.414)	0.218 ± 0.144 (0.195–0.242)	1.539 ± 0.026 (1.539–1.540)	99.059 ± 3.024 (99.017–99.101)

**Table 3:** MM-PBSA binding energy components calculated for the selected protein–ligand complexes of MMP2. All energy values are expressed in kcal/mol as the average ± standard error of the mean (SEM).

System	ΔE_vdw_	ΔE_ele_	ΔE_pb_	ΔE_surf_	ΔE_gas_	ΔG_solv_	ΔH_total_
09	−43.50 ± 0.12	−14.54 ± 0.19	33.58 ± 0.20	−4.83 ± 0.01	−58.04 ± 0.24	28.75 ± 0.20	−29.29 ± 0.13
I52	−46.25 ± 0.11	−8.51 ± 0.22	27.98 ± 0.21	−4.45 ± 0.01	−54.76 ± 0.26	23.52 ± 0.20	−31.24 ± 0.14
S1005	−29.55 ± 0.23	−18.78 ± 0.40	32.23 ± 0.39	−3.39 ± 0.02	−48.33 ± 0.51	28.84 ± 0.38	−19.49 ± 0.19
S2788	−32.60 ± 0.11	−18.48 ± 0.13	34.79 ± 0.19	−3.39 ± 0.01	−51.08 ± 0.19	31.40 ± 0.18	−19.68 ± 0.10
S2807	−13.48 ± 0.39	−3.33 ± 0.28	12.21 ± 0.44	−1.78 ± 0.05	−16.81 ± 0.55	10.44 ± 0.40	−6.37 ± 0.20
S1178	−41.47 ± 0.10	−8.36 ± 0.20	25.04 ± 0.23	−4.01 ± 0.01	−49.83 ± 0.23	21.02 ± 0.23	−28.81 ± 0.12
S7397	−50.75 ± 0.14	−14.95 ± 0.21	40.56 ± 0.24	−4.95 ± 0.01	−65.70 ± 0.24	35.62 ± 0.23	−30.09 ± 0.16

**Note**: ΔE_vdw_ van der Waals energy, ΔE_ele_ electrostatic energy, ΔE_pb_ polar solvation energy, ΔE_surf_ nonpolar solvation energy. $∆E_{gas}=∆E_{vdw}+∆E_{ele}$, $∆G_{solv}=∆E_{pb}+∆E_{surf}$, and $∆H_{solv}=∆E_{gas}+∆G_{solv}$.

**Table 4:** Docking scores of the best five FDA-approved compounds complexed with MMP3, along with the lead compound (09), co-crystallized ligand (L04) and the reference (ref) compound.

Mol_ID	Name	Docking Score (Kcal/mol)
S2788	Capmatinib	−11.5
S1178	Regorafenib	−11.4
S7397	Sorafenib	−11.0
S1005	Axitinib	−10.9
S2807	Dabrafenib	−9.4
09	4-(3-(2,4-dimethylphenyl)-5-(naphthalen-1-yl)-4,5-dihydro-1H-pyrazol-1-yl)benzenesulfonamide	−9.7
L04	6-(4′-Fluoro-Biphenyl-4-Yl)-4-(3-Methyl-1-Phenylcarbamoyl-Butylcarbamoyl)-2-[4-(1-Oxo-1,3-Dihydro-Isoindol-2-Yl)-Butyl]-Hexanoic Acid	−11.4
Ref	N-Hydroxy-2-((4-(4-(6-(2-hydroxyethoxy)pyridin-2-yl)-3-methylphenyl)piperidin-1-yl)sulfonyl)-2-methylpropanamide	−10.6

**Table 5:** Calculated average parameters over a 200 ns MD simulation for all complexes of MMP3 along with 95% confidence intervals.

System	RMSD (nm)	RMSF (nm)	Rg (nm)	SASA (nm^2^)
MMP3-apo	0.246 ± 0.038 (0.246–0.247)	0.125 ± 0.083 (0.112–0.138)	1.498 ± 0.014 (1.498–1.498)	95.456 ± 3.003 (95.414–95.498)
MMP3-L04	0.186 ± 0.028 (0.185–0.186)	0.106 ± 0.068 (0.095–0.116)	1.497 ± 0.010 (1.497–1.498)	96.249 ± 1.944 (96.223–96.276)
MMP3-Ref	0.200 ± 0.025 (0.199–0.200)	0.096 ± 0.068 (0.086–0.107)	1.502 ± 0.008 (1.502–1.502)	95.066 ± 1.842 (95.041–95.092)
MMP3-S1178	0.245 ± 0.033 (0.245–0.246)	0.097 ± 0.057 (0.088–0.105)	1.491 ± 0.010 (1.490–1.491)	95.558 ± 1.964 (95.531–95.586)
MMP3-S2788	0.199 ± 0.016 (0.199–0.200)	0.093 ± 0.051 (0.085–0.101)	1.476 ± 0.010 (1.476–1.476)	93.854 ± 2.261 (93.823–93.886)

**Table 6:** MM-PBSA binding energy components calculated for the selected protein–ligand complexes of MMP3 complexes. All energy values are expressed in kcal/mol as the average ± standard error of the mean (SEM).

System	ΔE_vdw_	ΔE_ele_	ΔE_pb_	ΔE_surf_	ΔE_gas_	ΔG_solv_	ΔH_total_
Ref	−57.51 ± 0.10	−11.79 ± 0.19	42.28 ± 0.18	−5.25 ± 0.00	−69.31 ± 0.21	37.03 ± 0.18	−32.28 ± 0.14
S1178	−32.12 ± 0.19	−18.34 ± 0.30	37.18 ± 0.34	−3.41 ± 0.01	−50.47 ± 0.42	33.77 ± 0.33	−16.70 ± 0.13
S2788	−59.97 ± 0.10	−8.07 ± 0.24	41.41 ± 0.23	−4.98 ± 0.00	−68.04 ± 0.26	36.43 ± 0.23	−31.61 ± 0.14

**Note**: ΔE_vdw_ van der Waals energy, ΔE_ele_ electrostatic energy, ΔE_pb_ polar solvation energy, ΔE_surf_ nonpolar solvation energy. $∆E_{gas}=∆E_{vdw}+∆E_{ele}$, $∆G_{solv}=∆E_{pb}+∆E_{surf}$, and $∆H_{solv}=∆E_{gas}+∆G_{solv}$.

**Table 7:** Clinical and pharmacological characteristics of the five prioritized FDA-approved drugs.

Internal ID	Generic Name	Drug Class/ Principal Targets	FDA-Approved Indication(s)	Major Safety Considerations	Relevant Clinical Pharmacokinetics
S1178	Regorafenib	Multikinase inhibitor; VEGFR1-3, TIE2, KIT, RET, RAF-1/BRAF, PDGFRα/β, FGFR1/2 and others	Previously treated metastatic colorectal cancer; GIST after imatinib and sunitinib; HCC after Sorafenib	Boxed warning for hepatotoxicity; hypertension, hemorrhage, and hand–foot skin reaction	160 mg once daily on days 1–21 of each 28-day cycle; 99.5% plasma-protein-bound; t1/2 ≈ 28 h; steady-state Cmax = 3.9 µg/mL (8.1 µM total) [31]
S7397	Sorafenib	Multikinase inhibitor; CRAF/BRAF, VEGFR1-3, PDGFRβ, KIT, FLT3 and RET	Unresectable HCC; advanced RCC; progressive radioactive-iodine-refractory differentiated thyroid carcinoma	Hand–foot skin reaction, hypertension, hemorrhage, and drug-induced liver injury	400 mg twice daily; 99.5% plasma-protein-bound; t1/2 = 25–48 h [32]
S1005	Axitinib	VEGFR tyrosine-kinase inhibitor; principally VEGFR1-3	Advanced RCC, alone after prior systemic therapy or in approved first-line combinations	Hypertension, thromboembolic and hemorrhagic events, cardiac failure, and hepatotoxicity	5 mg twice daily; >99% plasma-protein-bound; t1/2 = 2.5–6.1 h [33]
S2807	Dabrafenib	BRAF inhibitor, particularly BRAF V600 mutant kinases	BRAF V600 mutant melanoma; with trametinib for selected BRAF V600E positive malignancies including NSCLC, anaplastic thyroid cancer, other solid tumors, and pediatric low-grade glioma	Pyrexia, new primary malignancies, hemorrhage, and serious skin toxicities	Adult dose 150 mg twice daily; 99.7% plasma-protein-bound; t1/2 = 8 h [34]
S2788	Capmatinib	Selective MET tyrosine-kinase inhibitor	Metastatic NSCLC with MET exon 14 skipping mutation	ILD/pneumonitis, hepatotoxicity, pancreatic toxicity, and edema	400 mg twice daily; 96% plasma-protein-bound; t1/2 = 6.5 h; reported steady-state Cmax = 4.78 µg/mL (11.6 µM total) under fasting conditions [35]

## Data Availability

The data supporting the findings of this study are available in Supplementary Materials.

## Supplementary Materials

The following supporting information can be downloaded at: https://www.mdpi.com/article/10.3390/ijms27177756/s1.

## Abbreviations

The following abbreviations are used in this manuscript:

IPF	Idiopathic pulmonary fibrosis
MMP2	Matrix metalloproteinase-2
FDA	Food and Drug Administration
MD	Molecular dynamics
MMP3	Matrix metalloproteinase-3
ECM	Extracellular matrix
EMT	Epithelial–mesenchymal transition
TGF-β	Transforming growth factor-beta
VS	Virtual screening
H	Hydrophobic
Ar	Aromatic
HBA	Hydrogen bond acceptor
HBD	Hydrogen bond donor
ROC	Receiver Operating Characteristic
TP	True positive
FP	False positive
GH	Goodness-of-hit
EF	Enrichment Factor
I52	1HOV co-crystallized ligand
RMSD	Root Mean Square Deviation
AUC	Area under curve
TG	Total Gain
RIE	Robust Initial Enhancement
BEDROC	Boltzmann-Enhanced Discrimination of Receiver Operating Characteristic
RMSF	Root mean square fluctuation
Rg	Radius of gyration
SASA	Solvent-accessible surface area
L04	1HFS co-crystallized ligand
MMFF94	Merck Molecular Force Field 94
DUD-E	A Database of Useful Decoys-Enhanced
SMILES	Simplified Molecular Input Line Entry System
TPR	True positive rate
TNR	True negative rate
ACC	Accuracy
Y_a_	Number of actives retrieved within the top-ranked fraction of the dataset
FN	False negative
TN	True negative
A	Total number of active compounds
D	Total number of inactive compounds
T	Total number of compounds in the validation dataset
H_a_	Number of active compounds retrieved as hits
H_t_	Total number of hits retrieved
