# Supplementary material for: Computational Repurposing of FDA-Approved Drugs as Candidate MMP2 Inhibitors with Putative MMP3 Cross-Activity
Source: Int J Mol Sci. 2026 Aug 29;27(17):7756. doi: 10.3390/ijms27177756 (PMC13565895; doi:10.3390/ijms27177756)
Supplement: Supplementary file 1 [file ijms-27-07756-s001.zip › Supplementary Material_VF.pdf]

Supplementary Information for

Computational repurposing of FDA-approved drugs as candidate MMP2 inhibitors with putative MMP3 cross-activity

Saad Zekri [1], Nouhaila Ait Lahcen [1], Wissal Liman [1], Francesca Bianchini [2], Mehdi Oubahmane [3,*], Ismail Hdoufane [1,*], Driss Cherqaoui [1]

[1] Cadi Ayyad University, UCA, Faculty of Sciences Semlalia, Laboratory of Molecular Chemistry, B.P. 2390, 40000 Marrakech, Morocco.
[2] Department of Experimental and Clinical Biomedical Sciences "Mario Serio", Section of Experimental Pathology and Oncology, University of Florence, 50141 Florence, Italy
[3] Mohammed VI Polytechnic University (UM6P), Hay Moulay Rachid, 43150 Ben Guerir, Morocco.
* Correspondence: oubahmanem@gmail.com; i.hdoufane@uca.ac.ma

Table S1. Chemical Structures, pIC_{50} Values, Pharmacophore-fit score and matched features in training and test set of Sulfonamide Derivatives for MMP-2 Database.

ID	Structure	pIC_{50}	Pharmacophore-Fit score	Matched Features pairs
01	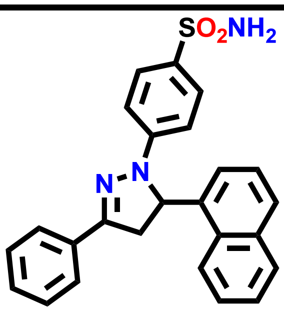	5.10	108.69	10
02	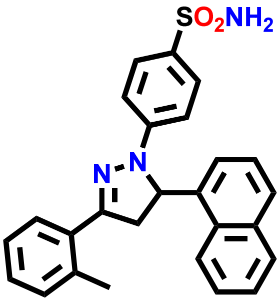	5.54	106.90	10

03	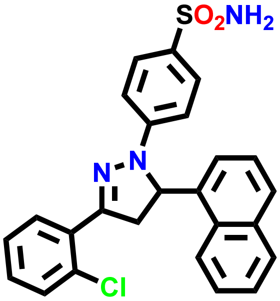	5.06	108.54	10
04	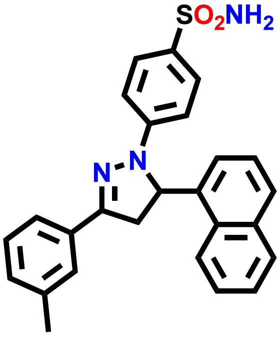	5.49	108.64	10
05	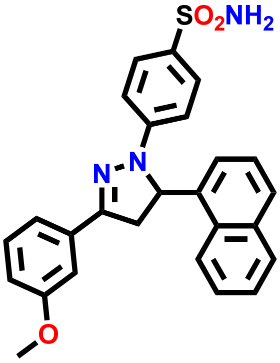	5.32	108.66	10
06	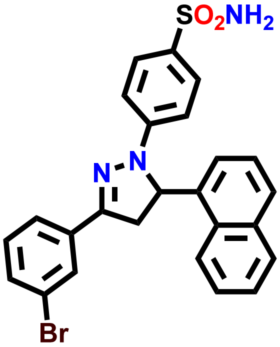	4.94	108.60	10
07	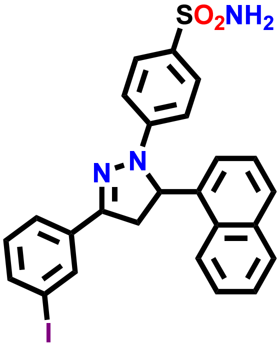	5.03	108.60	10

08	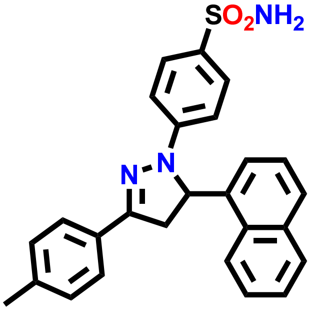	5.17	108.68	10
09	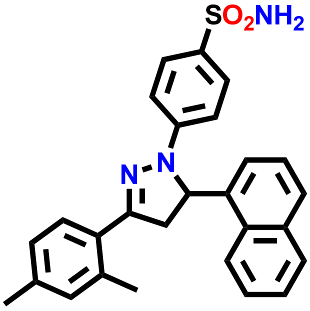	6.68	107.69	10
10	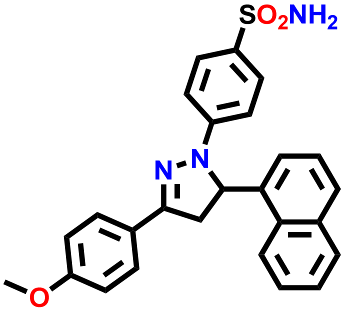	5.37	108.69	10
11	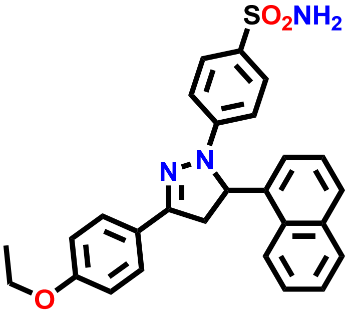	6.09	108.66	10
12	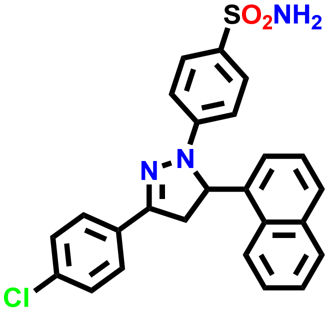	5.57	108.65	10

13	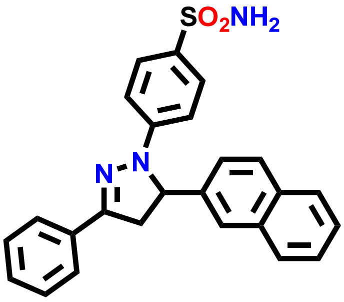	4.90	108.90	10
14	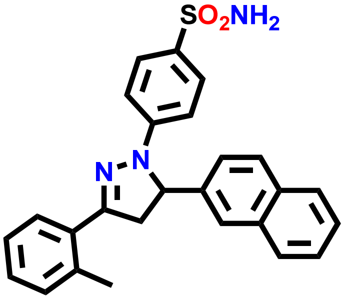	4.94	98.64	10
15	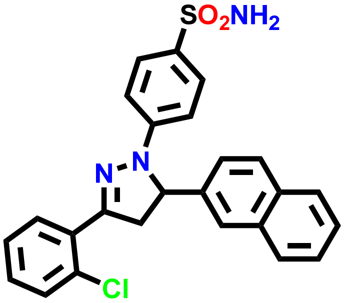	4.83	108.66	10
16	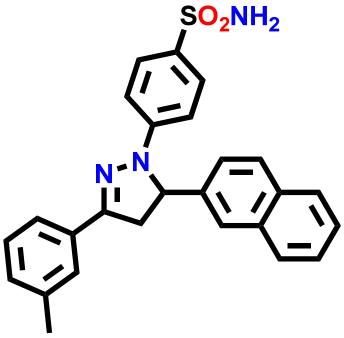	5.41	108.88	10
17	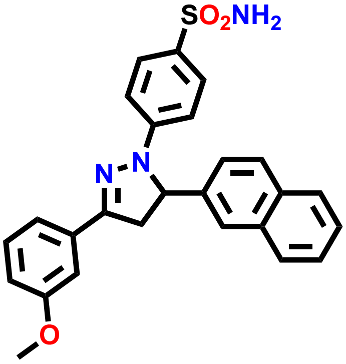	5.67	108.89	10

18	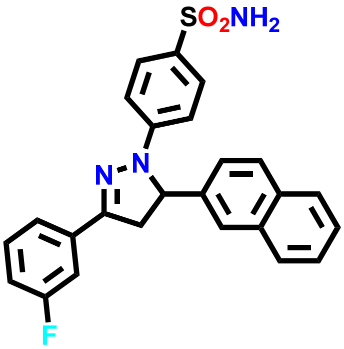	4.62	108.87	10
19	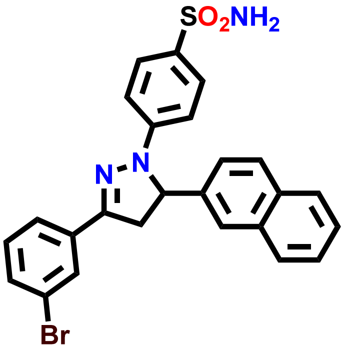	4.75	108.75	10
20	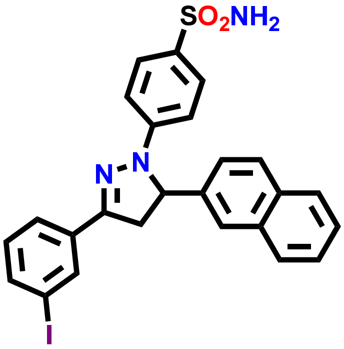	4.80	108.88	10
21	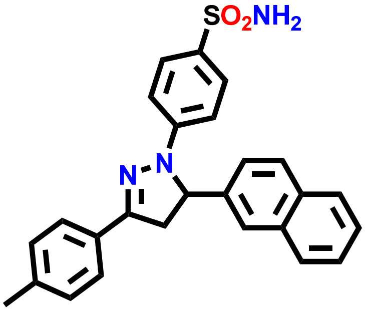	5.55	108.85	10
22	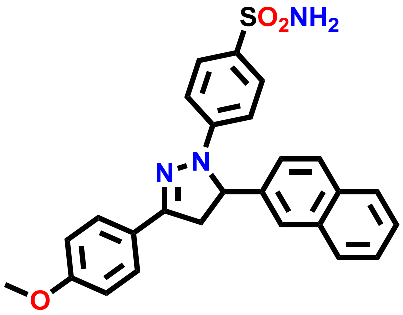	5.00	108.67	10

23	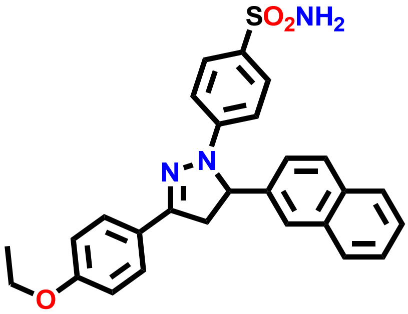	4.83	108.86	10
24	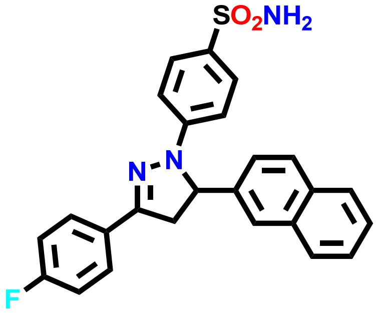	4.77	108.85	10
25	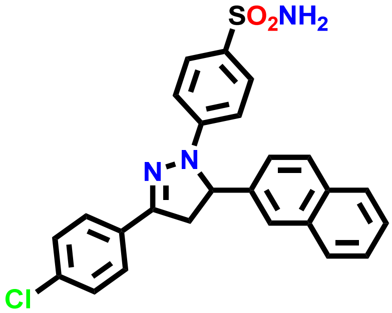	5.44	108.85	10

Table S2. Statistical parameters used for the validation of the pharmacophore model, including sensitivity, specificity, AUC, EF, GH, and retrieved hits.

TP	25
TN	1247
FP	3
FN	0
A	25
D	1250
Sensitivity (True positive rate)	1
Specificity (True negative rate)	0.998
AUC	1
ACC	0.998

GH	0.917
EF	45.5

Table S3. Chemical structures of the selected FDA-approved compounds in both targets, along with the reference and the co-crystallized ligands.

Mol_ID	Structure
09 (Lead compound)	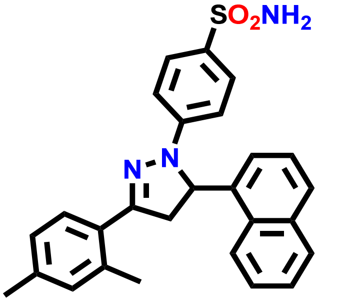
I52	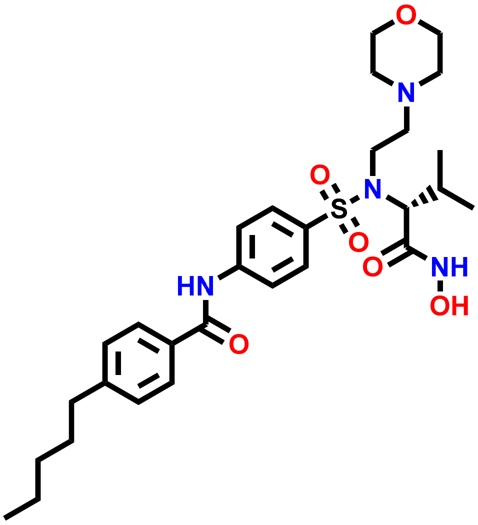
S1005	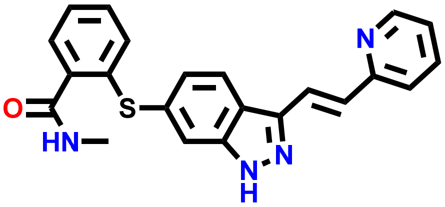
S1178	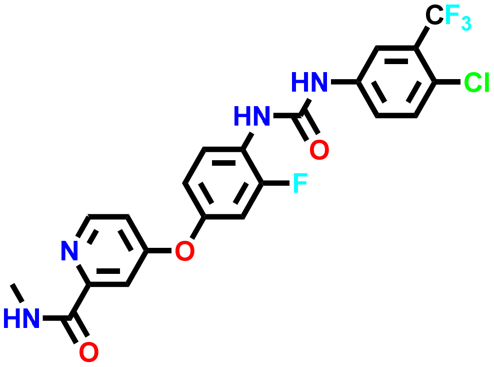

S2788	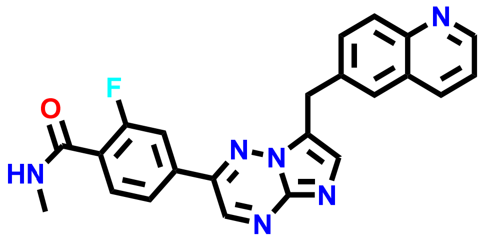
S2807	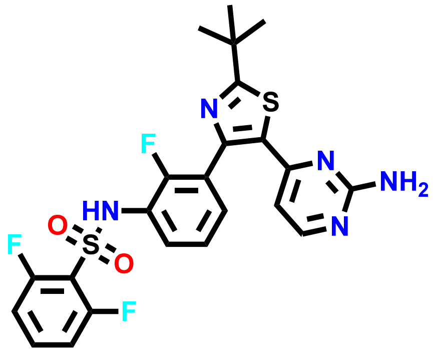
S7397	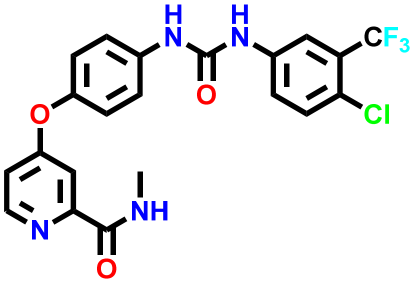
Ref	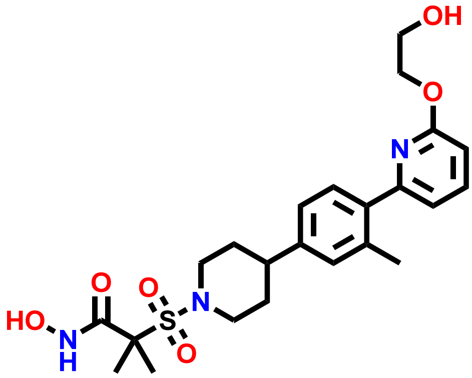
L04	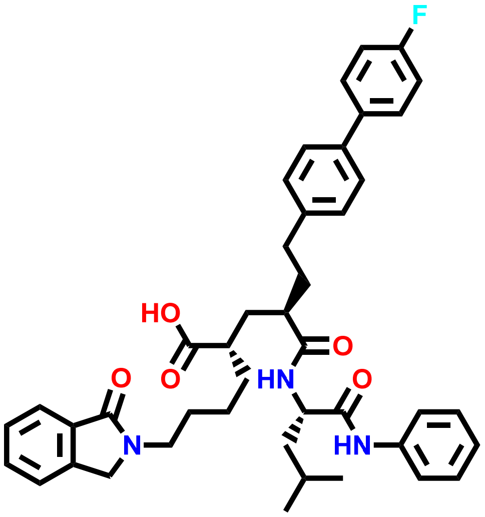

Table S4. Molecular docking interactions of the top five ligands, lead and co-crystallized compounds with the key binding site residues of 1HOV target.

Cmpd.	Category	Type	Residues	Distance (Å)
09 (Lead compound)	Hydrogen Bond	Conventional HB	A:ALA86	2.2465
	Electrostatic	Pi-Cation	A:ZN166	4.8562
	Electrostatic	Pi-Cation	A:ZN166	4.7608
	Hydrophobic	Pi-Pi Stacked	A:HIS120	4.7847
	Hydrophobic	Pi-Pi Stacked	A:HIS120	3.9955
	Hydrophobic	Pi-Pi T-shaped	A:TYR74	5.6322
	Hydrophobic	Pi-Pi T-shaped	A:HIS124	5.5355
	Hydrophobic	Amide-Pi Stacked	A:GLY81;LEU82	4.1576
	Hydrophobic	Pi-Alkyl	A:TYR74	4.6745
	Hydrophobic	Pi-Alkyl	A:LEU83	4.4857
I52 (Co-crystallized)	Hydrogen Bond	Conventional HB	A:LEU83	1.7321
	Hydrogen Bond	Conventional HB	A:ALA84	2.3754
	Hydrogen Bond	Conventional HB	A:ALA84	2.3248
	Hydrogen Bond	Conventional HB	A:GLU121	2.3888
	Hydrogen Bond	Carbon HB	A:ALA84	3.6515
	Other	Metal-Acceptor	A:ZN166	2.6168
	Hydrogen Bond	Pi-Donor HB	A:THR143	2.9479
	Hydrophobic	Pi-Sigma	A:LEU83	3.8983
	Hydrophobic	Alkyl	A:LEU137	4.5098
	Hydrophobic	Pi-Alkyl	A:LEU116	5.4108
	Hydrophobic	Pi-Alkyl	A:LEU137	4.8700
S1005	Hydrogen Bond	Conventional HB	A:ALA136	2.4457
	Hydrogen Bond	Carbon HB	A:GLY135	3.5663
	Hydrogen Bond	Carbon HB	A:LEU150	3.7935
	Hydrogen Bond	Pi-Donor HB	A:HIS120	3.1487
	Hydrogen Bond	Pi-Donor HB	A:THR143	2.9771
	Hydrophobic	Pi-Sigma	A:LEU83	3.8184
	Hydrophobic	Pi-Sigma	A:LEU137	3.9976
	Hydrophobic	Pi-Sigma	A:LEU137	3.8238
	Hydrophobic	Pi-Sigma	A:LEU137	3.9600
	Hydrophobic	Pi-Pi Stacked	A:HIS120	5.1702
	Hydrophobic	Pi-Pi T-shaped	A:TYR142	5.5045
	Hydrophobic	Pi-Pi T-shaped	A:PHE148	5.1520
	Hydrophobic	Pi-Alkyl	A:ALA139	5.1828
	Hydrophobic	Pi-Alkyl	A:LEU116	5.2868
	Hydrophobic	Pi-Alkyl	A:LEU137	4.6338
	Hydrophobic	Pi-Alkyl	A:ALA119	4.6292

S1178	Hydrogen Bond	Conventional HB	A:ILE141	2.4859
	Hydrogen Bond	Conventional HB	A:ILE141	2.1478
	Hydrogen Bond	Carbon HB	A:ARG149	3.4622
	Other	Metal-Acceptor	A:ZN166	2.4983
	Halogen	Halogen (Fluorine)	A:PRO134	3.6840
	Halogen	Halogen (Fluorine)	A:GLY135	3.6757
	Halogen	Halogen (Fluorine)	A:ALA136	3.1234
	Halogen	Halogen (Fluorine)	A:ALA136	3.0893
	Halogen	Halogen (Fluorine)	A:ALA139	3.4525
	Halogen	Halogen (Fluorine)	A:PRO140	3.2395
	Halogen	Halogen (Fluorine)	A:ILE141	3.1615
	Electrostatic	Pi-Cation	A:ZN166	4.9463
	Hydrophobic	Pi-Sigma	A:THR143	3.9428
	Hydrophobic	Pi-Pi Stacked	A:HIS120	4.0765
	Hydrophobic	Pi-Alkyl	A:LEU83	5.1556
	Hydrophobic	Pi-Alkyl	A:LEU137	4.7846
S2788	Hydrogen Bond	Carbon HB	A:PRO140	3.4950
	Hydrogen Bond	Carbon HB	A:VAL117	3.4927
	Electrostatic	Pi-Cation	A:ZN166	4.4702
	Hydrophobic	Pi-Sigma	A:LEU83	3.9536
	Hydrophobic	Pi-Pi Stacked	A:HIS120	4.4311
	Hydrophobic	Pi-Pi Stacked	A:HIS120	3.7800
	Hydrophobic	Pi-Alkyl	A:ALA84	4.9141
	Hydrophobic	Pi-Alkyl	A:LEU82	4.1580
	Hydrophobic	Pi-Alkyl	A:LEU83	5.1465
S2807	Hydrogen Bond	Conventional HB	A:ALA84	2.2701
	Other	Metal-Acceptor	A:ZN166	2.7738
	Halogen	Halogen (Fluorine)	A:ALA84	3.1566
	Halogen	Halogen (Fluorine)	A:GLU121	3.4822
	Hydrophobic	Pi-Sigma	A:LEU83	3.9297
	Hydrophobic	Pi-Sigma	A:TYR74	3.7803
	Other	Pi-Sulfur	A:HIS120	3.9465
	Hydrophobic	Pi-Pi Stacked	A:HIS120	4.3425
	Hydrophobic	Pi-Pi Stacked	A:HIS130	5.1525
	Hydrophobic	Pi-Pi T-shaped	A:HIS124	5.2096
	Hydrophobic	Alkyl	A:LEU82	5.0285
	Hydrophobic	Pi-Alkyl	A:TYR74	4.4288
	Hydrophobic	Pi-Alkyl	A:ALA84	5.0569
S7397	Hydrogen Bond	Conventional HB	A:LEU83	2.6971
	Hydrogen Bond	Conventional HB	A:ILE141	2.6343
	Hydrogen Bond	Conventional HB	A:ILE141	2.1087
	Hydrogen Bond	Conventional HB	A:ALA84	2.5688
	Hydrogen Bond	Conventional HB	A:GLU121	2.5634
	Hydrogen Bond	Carbon HB	A:ARG149	3.5458
	Hydrogen Bond	Carbon HB	A:GLU121	3.5287
	Halogen	Halogen (Fluorine)	A:PRO134	3.6878
	Halogen	Halogen (Fluorine)	A:ALA136	3.0525
	Halogen	Halogen (Fluorine)	A:ALA136	3.1322
	Hydrophobic	Pi-Sigma	A:THR143	3.9766
	Hydrophobic	Pi-Sigma	A:HIS124	3.6212

Hydrophobic	Pi-Pi T-shaped	A:TYR142	5.4602
Hydrophobic	Pi-Alkyl	A:LEU83	5.0032
Hydrophobic	Pi-Alkyl	A:LEU137	4.7530

Table S5. Molecular docking interactions of the top two ligands, refernce and co-crystallized compounds with the key binding site residues of 1HFS target.

Cmpd.	Category	Type	Residues	Distance (Å)
Reference	Hydrogen Bond	Conventional HB	A:LEU164	1,8313
	Hydrogen Bond	Conventional HB	A:ALA165	2,4419
	Hydrogen Bond	Conventional HB	A:TYR223	2,8172
	Hydrogen Bond	Conventional HB	A:ARG233	2,0558
	Hydrogen Bond	Conventional HB	A:ASN162	2,2184
	Hydrogen Bond	Conventional HB	A:ASN162	2,2162
	Hydrogen Bond	Carbon HB	A:LEU218	3,3090
	Hydrogen Bond	Carbon HB	A:LEU222	3,6528
	Electrostatic	Pi-Cation	A:HIS224	4,6809
	Hydrophobic	Pi-Pi Stacked	A:HIS201	4,5000
	Hydrophobic	Pi-Pi Stacked	A:HIS224	4,7331
	Hydrophobic	Pi-Pi T-shaped	A:TYR223	5,0638
	Hydrophobic	Pi-Alkyl	A:LEU197	4,1696
	Hydrophobic	Pi-Alkyl	A:LEU226	5,4716
	Hydrophobic	Pi-Alkyl	A:VAL198	5,1993
L04 (Co-crystallized)	Electrostatic;Other	Attractive Charge;Metal-Acceptor	A:ZN257	2,4056
	Hydrogen Bond	Conventional HB	A:LEU164	2,0348
	Hydrogen Bond	Conventional HB	A:TYR223	1,9883
	Hydrogen Bond	Carbon HB	A:ASN162	3,5792
	Other	Metal-Acceptor	A:ZN257	2,5415
	Hydrophobic	Pi-Sigma	A:LEU218	3,8103
	Hydrophobic	Pi-Pi Stacked	A:HIS166	4,7106
	Hydrophobic	Pi-Pi Stacked	A:HIS201	4,0642
	Hydrophobic	Pi-Pi T-shaped	A:TYR155	4,9913
	Hydrophobic	Pi-Pi T-shaped	A:TYR168	5,6716
	Hydrophobic	Pi-Pi T-shaped	A:TYR223	5,2662
	Hydrophobic	Pi-Pi T-shaped	A:TYR223	4,9913
	Hydrophobic	Alkyl	A:VAL163	4,3633
	Hydrophobic	Alkyl	A:LEU222	5,1169
	Hydrophobic	Pi-Alkyl	A:VAL198	5,0386
	Hydrophobic	Pi-Alkyl	A:LEU197	4,9286
	Hydrophobic	Pi-Alkyl	A:LEU164	5,2207
S1178	Halogen	Halogen (Fluorine)	A:HIS201	3,0799
	Halogen	Halogen (Fluorine)	A:HIS201	3,2978
	Halogen	Halogen (Fluorine)	A:GLU202	3,3864
	Halogen	Halogen (Fluorine)	A:HIS211	3,1437
	Halogen	Halogen (Fluorine)	A:PRO221	3,3502
	Halogen	Halogen (Fluorine)	A:PRO221	2,7116
	Halogen	Halogen (Fluorine)	A:HIS224	2,8390
	Electrostatic	Pi-Cation	A:HIS224	4,2378
	Hydrophobic	Pi-Pi Stacked	A:HIS201	4,1228
	Hydrophobic	Pi-Pi Stacked	A:HIS224	4,4103

	Hydrophobic	Pi-Alkyl	A:HIS201	4,1478
	Hydrophobic	Pi-Alkyl	A:HIS211	5,2117
	Hydrophobic	Pi-Alkyl	A:LEU226	5,1076
	Hydrophobic	Pi-Alkyl	A:LEU197	5,1636
	Hydrophobic	Pi-Alkyl	A:LEU218	5,3368
	Hydrophobic	Pi-Alkyl	A:LEU164	5,4745
	Hydrophobic	Pi-Alkyl	A:VAL198	4,6757
S2788	Hydrogen Bond	Carbon HB	A:VAL163	3,6664
	Electrostatic	Pi-Cation	A:ZN257	3,8537
	Hydrogen Bond	Pi-Donor HB	A:LEU164	2,7442
	Hydrophobic	Pi-Pi Stacked	A:HIS201	4,5358
	Hydrophobic	Pi-Pi Stacked	A:HIS201	3,7228
	Hydrophobic	Pi-Pi Stacked	A:HIS205	4,0863
	Hydrophobic	Pi-Pi T-shaped	A:TYR223	5,1416
	Hydrophobic	Pi-Alkyl	A:VAL163	4,8700
	Hydrophobic	Pi-Alkyl	A:VAL163	5,0279
	Hydrophobic	Pi-Alkyl	A:LEU164	4,7280
	Hydrophobic	Pi-Alkyl	A:VAL198	5,4577
	Hydrophobic	Pi-Alkyl	A:VAL198	5,0258
